# The Ammann–Kramer–Neri tiling model of a P-ZnMgEr Bergman-type quasicrystal based on in-house X-ray diffraction

**DOI:** 10.1107/S205252062600003X

**Published:** 2026-02-10

**Authors:** Ireneusz Buganski, Stanislav Vrtnik, Andreja Jelen, Jože Luzar, Radoslaw Strzalka, Janusz Wolny, Joachim Kusz, Nobuhisa Fujita

**Affiliations:** a Faculty of Physics and Applied Computer Science AGH University of Krakow Al. Mickiewicza 30 Krakow 30-059 Poland; b Jožef Stefan Institute Jamova 39 Ljubljana SI-1000 Slovenia; c Faculty of Mathematics and Physics University of Ljubljana Jadranska 19 Ljubljana SI-1000 Slovenia; d Institute of Physics University of Silesia 75 Pułku Piechoty 1a Katowice 41-500 Poland; e Institute of Multidisciplinary Research for Advanced Materials Tohoku University Sendai 980-8577 Japan; University of Geneva, Switzerland

**Keywords:** quasicrystals, structure solution, Bergman phase, Ammann–Kramer–Neri tiling

## Abstract

This study presents the atomic structure solution of a primitive icosahedral Zn_70.83_Mg_20.31_Er_8.86_ quasicrystal using in-house X-ray diffraction and the Ammann–Kramer–Neri tiling framework. The structural model with 344 parameters refined against 2674 Bragg peaks achieves a reasonable *R* factor of 0.1407. Both Bergman and Tsai clusters, key motifs in the quasicrystal architecture, are identified within the structure, suggesting a hybrid character that bridges traditionally distinct quasicrystal types. The Tsai clusters show positional disorder and unusually short Mg–Mg distances (∼2.3 Å), hinting at the complex nature of the bond. Despite only moderate data resolution, the model reveals phason-related features and relatively low phasonic disorder, consistent with previously modelled Zn–Mg–Tm. This work demonstrates that the tiling can reliably reproduce real atomic configurations, which supports the use of models based on the Ammann–Kramer–Neri tiling in quasicrystalline atomic structure research. The findings contribute to ongoing efforts to decode the structural diversity and physical properties of rare-earth-containing quasicrystals.

## Abstract 2

A structural solution is presented of a primitive icosahedral ZnMgEr quasicrystal representing the Bergman-type family. The structure model is based on atomic decoration of the Ammann–Kramer–Neri tiling.

## 1. Introduction

Icosahedral quasicrystals in the Zn–Mg–rare earth (RE) system form an important subgroup of Bergman-type quasicrystals. They occur in both the primitive (P) and face-centred (F) icosahedral lattices (Luo *et al.*, 1993 ▸; Niikura *et al.*, 1994*a* ▸; Niikura *et al.*, 1994*b* ▸; Tsai *et al.*, 1994 ▸). The F-type phase, which forms large single crystals, typically has the composition Zn_56.8_Mg_34.6_RE_8.7_ (RE = Y, Er, Ho, Dy, Tb) (Fisher *et al.*, 1998 ▸). The P-type phase occurs at Zn_76_Mg_14_RE_10_ (RE = Ho, Er, Tm) (Uhrig *et al.*, 2003 ▸; Uhrig *et al.*, 2005 ▸), reflecting a higher Zn concentration. Under specific growth conditions, an F-type Zn_62_Mg_29_Y_9_ phase with a composition different from the other family members can also be obtained (Langsdorf & Assmus, 1998 ▸). In addition to the icosahedral phases, decagonal quasicrystals occur near Zn_58_Mg_40_RE_2_ (RE = Dy, Ho, Er, Tm, Lu) (Sato *et al.*, 1997 ▸; Singh *et al.*, 1998 ▸), highlighting the rich structural diversity within this chemical family.

The discovery of quasicrystals containing RE elements initiated strong interest in their magnetic properties, which might reflect the influence of quasiperiodicity. To date, Bergman-type quasicrystals are known to exhibit only spin-glass behaviour (Fisher *et al.*, 1999 ▸; Dolinšek *et al.*, 2001 ▸; Sato, 2005 ▸), whereas long-range magnetic order has been identified in certain Tsai-type phases, including the ferromagnetic Au_65_Ga_20_(Gd, Tb)_15_ (Tamura *et al.*, 2021 ▸) and the antiferromagnetic Au_56_In_28.5_Eu_15.5_ (Tamura *et al.*, 2025 ▸).

From an atomic structure perspective, Tsai- and Bergman-type phases are closely related. Li *et al.* (2008 ▸) first demonstrated that pseudo-Bergman clusters effectively describe the structure of the Tsai-type 2/1 Ag_42_In_42_Yb_16_ phase. Buganski & Wolny (2023 ▸) further explored these similarities in 1/1 approximants. A deeper understanding of structural parallels and distinctions between Bergman- and Tsai-type phases is important for future materials design, yet a major obstacle is the limited knowledge of the atomic structure of Bergman-type quasicrystals.

Thus far, the only Bergman-type quasicrystal model capable of reproducing X-ray diffraction data with high accuracy is based on the Ammann–Kramer–Neri tiling (AKNT) (Kramer & Neri, 1984 ▸). This model was applied to the P-type Zn_69.5_Mg_20.9_Tm_9.6_ phase, for which 3010 reflections with |*F*| > 3σ(|*F*|) from the irreducible reciprocal space were collected (Buganski *et al.*, 2020 ▸). The model incorporates two rhombohedral units, prolate (acute) and oblate (obtuse), with an edge length of 21.7 Å.

The same six-dimensional (6D) AKNT framework was applied to the Tsai-type P-Cd_5.7_Yb phase, where the occupation domain was constructed in two versions: unshifted, and shifted by one-half of the long body diagonal of the 6D unit cell, exploiting the apparent duality between Tsai- and Bergman-type structures (Buganski *et al.*, 2024*a* ▸). This duality arises because the centres of Tsai clusters correspond to the vertices of the rhombic triacontahedron (RT) shells of Bergman clusters. In 6D crystallography, the distance between such cluster centres equals the distance from an RT vertex to the 6D unit-cell centre. Both AKNT models yielded an *R* factor of 0.115, implying that the average structures are similar. Compared with the ZnMgTm model, the Cd_5.7_Yb model had a proper treatment of the inversion symmetry, reducing the number of adjustable parameters. Nonetheless, this Tsai-type model remains inferior to the model proposed by Takakura *et al.* (2007 ▸).

In this work, we apply the AKNT approach to determine the atomic structure of the P-type ZnMgEr quasicrystal, aiming to expand structural knowledge within this family. Diffraction data were collected using an in-house diffractometer. Compared with the physical-space models for Cd_5.7_Yb (Buganski *et al.*, 2024*a* ▸) and Zn_69.5_Mg_20.9_Tm_9.6_ (Buganski *et al.*, 2020 ▸), our model includes additional constraints: atoms located on shared edges or faces of rhombohedra are refined with the same parameters. In a real quasicrystal, the local atomic environment may depend on whether the interface between rhombohedra involves identical or different units. For example, in decagonite, a natural decagonal AlNiFe phase, the phason-flippable Al atom is present at the edge of the thick rhombus but absent from the thin rhombus (Buganski & Bindi, 2021 ▸). However, because the matching rules for AKNT are unknown (Lück, 1990 ▸; Hann *et al.*, 2016 ▸), we cannot determine the minimum set of local constraints needed to preserve AKNT ordering. We therefore apply the constraint uniformly to all atoms located on faces and edges. This simplifies the model at the possible cost of increasing the final *R* factor if the constraint is incorrect. Nonetheless, the constraint is desired here because the in-house diffraction data have lower resolution than synchrotron data, and reducing the number of refined parameters improves the peak-to-parameter ratio.

An alternative approach to modelling icosahedral quasicrystals, in which the minimal set of matching rules can be explicitly determined, was proposed by Kalugin & Katz (2019 ▸). However, it requires defining a set of vertex-decorated prototiles that has not yet been established for any real quasicrystal. As such, this approach diverges from the AKNT framework used here.

## 2. Experimental

**Figure 1:**
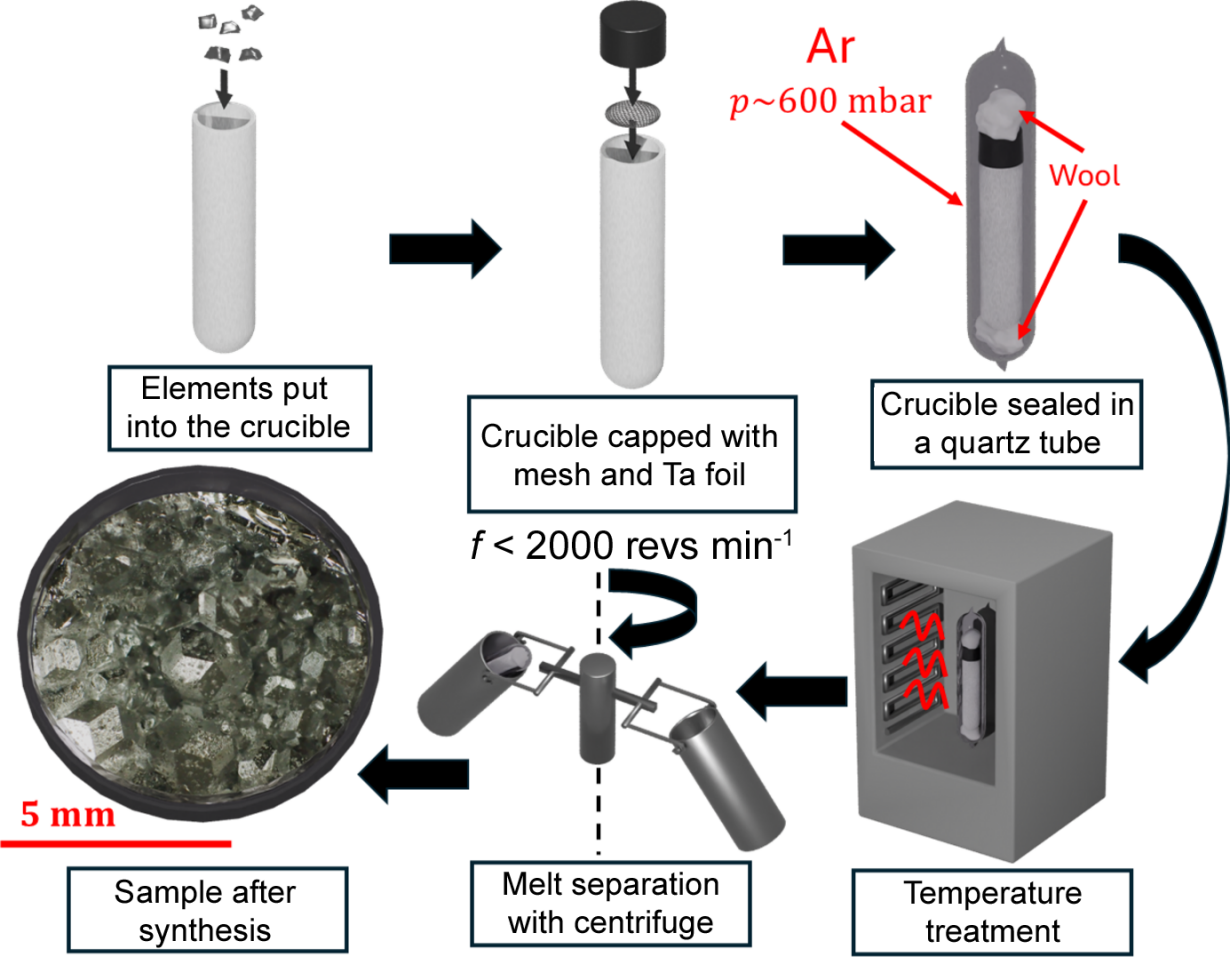
Sample synthesis process.

The crystal growth of single crystals was performed with the self-flux method, following the initial composition given by Uhrig *et al.* (2003 ▸). Zn (grains with <5 mm diameter with a purity of 99.999%), Mg (granules <2 mm diameter with a purity of 99.99%) and Er (pieces <3 mm diameter with a purity of 99.9%) were weighed in the initial composition of Zn_62.8_Mg_33.6_Er_3.6_ and placed in an alumina crucible. The total initial mass of the elements was 5 g. The crucible was capped with mesh and Ta foil and sealed under Ar in a quartz tube under a pressure of 600 mbar. Before applying Ar, the air was evacuated until a pressure of 3.1 × 10^−3^ Pa was reached. The sample was then placed in a muffle furnace for heat treatment. The sample was first heated to 750°C and kept at that temperature for 5 h, then slowly cooled to 590°C over a period of 80 h. The sample was annealed at 590°C for 76 h. Immediately after the sample was removed from the furnace, a centrifuge was used to separate the melt from the solidified phase. The rotation speed was 1500–2000 revs min^−1^ and was maintained for 20 min. Quartz wool packing was used to isolate the crucible from the quartz tube to prevent large breaks. After the process, large single crystals (∼1 mm in diameter) with RT morphology were observed under an optical microscope (Fig. 1 ▸).

**Figure 2:**
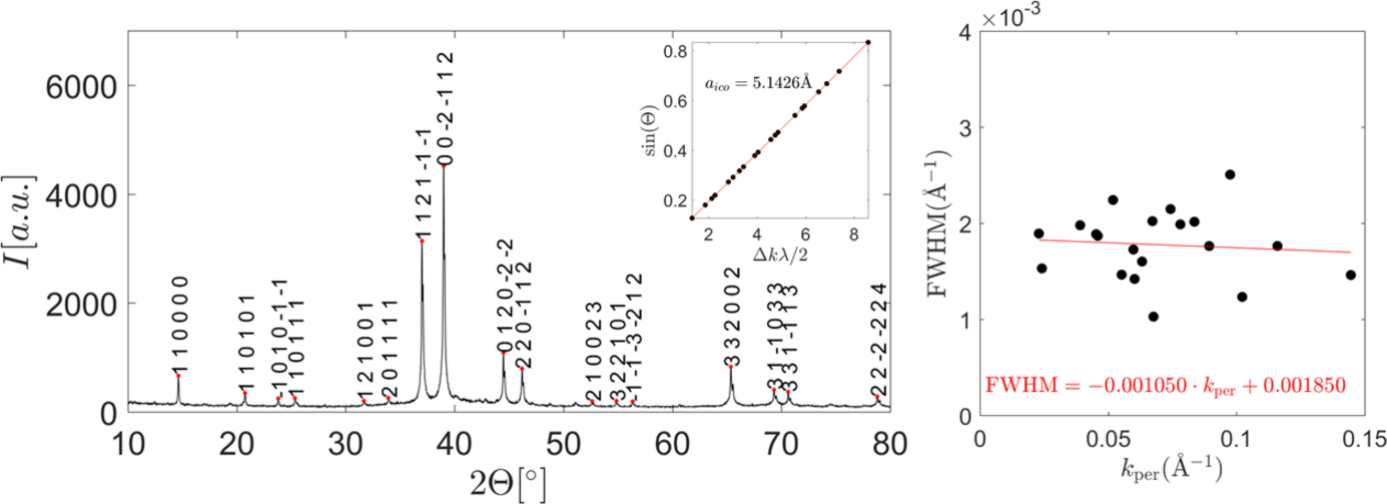
(Left) Indexed powder diffraction pattern with (inset) lattice constant fit and (right) a test of the presence of a linear phason strain.

A small part of the sample was ground in a mortar to obtain grains <25 µm for powder diffraction measurement with X-rays of Cu *K*α_1_ wavelength. All visible peaks could be indexed on a 6D basis using the Elser indexing scheme (Elser, 1986 ▸), as presented in Fig. 2 ▸. The lattice constant was fitted on the basis of a linear fit of the experimental and theoretical peak positions. The lattice constant expressed as an edge length of the rhombohedra in AKNT is *a*_ico_ = 5.1426 Å. No linear phase strain is seen in the full-width half-maximum (FWHM) plot as a function of the perpendicular space components of the reciprocal-space vector.

**Figure 3:**
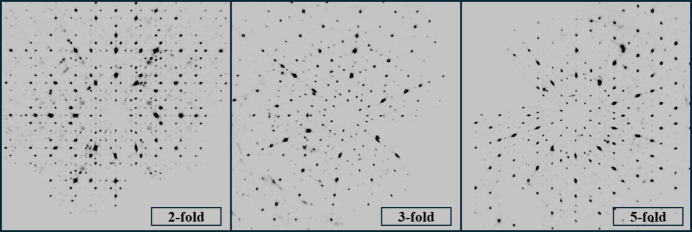
Unwarped diffraction images in planes perpendicular to the high-symmetry axes.

Single-crystal diffraction data were collected based on measurements of irregular grains with dimensions 90 × 80 × 50 µm. An Agilent SuperNova diffractometer with a four-circle goniometer was used with an Atlas CCD detector. The measurement was carried out at 100 K with an Mo lamp, with X-ray radiation of wavelength 0.71073 nm. The diffraction data were analysed with the *CrysAlisPRO* software (Agilent, 2014 ▸). In total, 448690 Bragg peaks were collected, among which 4790 are found in an irreducible part of reciprocal space with *R*_int_ = 0.149. For the refinement, only peaks with amplitude |*F*| > *u*(|*F*|) are taken, resulting in 2674 peaks. Data are deposited in the Open Science Framework (https://osf.io/8r5bd). Fig. 3 ▸ presents unwarped images from a single-crystal diffraction experiment presenting high-symmetry planes of reciprocal space. The diffuse scattering background is small, mostly accumulated in a plane perpendicular to the twofold symmetry axis. The lattice constant for the icosahedral structure is estimated to be *a*_ico_ = 5.1073 Å, smaller than the one estimated based on powder diffraction data.

The composition of the crystal was estimated with scanning electron microscopy/energy-dispersive X-ray spectroscopy (SEM/EDS). In Table S2 in the supporting information the measured compositions in chosen parts of the sample are shown, indicating how the content of Mg and Zn can vary by up to 2 at.%. The content of Er is more consistent in the sample grain, ranging from 8.51% to 9.14%. The average composition is concluded to be Zn_70.8_Mg_20.3_Er_8.9_. As the EDS maps indicate, the distribution of elements in the sample is uniform (Fig. S1).

## 3. *Ab initio* structure solution

The set of 2674 peaks from the irreducible part of reciprocal space was phased with the *SUPERFLIP* software (Palatinus & Chapuis, 2007 ▸). The final *R* factor converged to 0.33284. This is a high score compared with the data used for the structure solution of Cd_5.7_Yb (*R* = 0.167) (Takakura *et al.*, 2007 ▸) or Zn_69.5_Mg_20.9_Tm_9.6_ (*R* = 0.145) (Buganski *et al.*, 2020 ▸) – both P-type icosahedral quasicrystals. The lower the initial value of the *R* factor, the closer the solution is to the real atomic structure. It can be inferred that the data quality is lower than for the former structures and this correlates with the high value of *R*_int_ obtained during data integration.

### 3.1. Perpendicular space analysis

**Figure 4:**
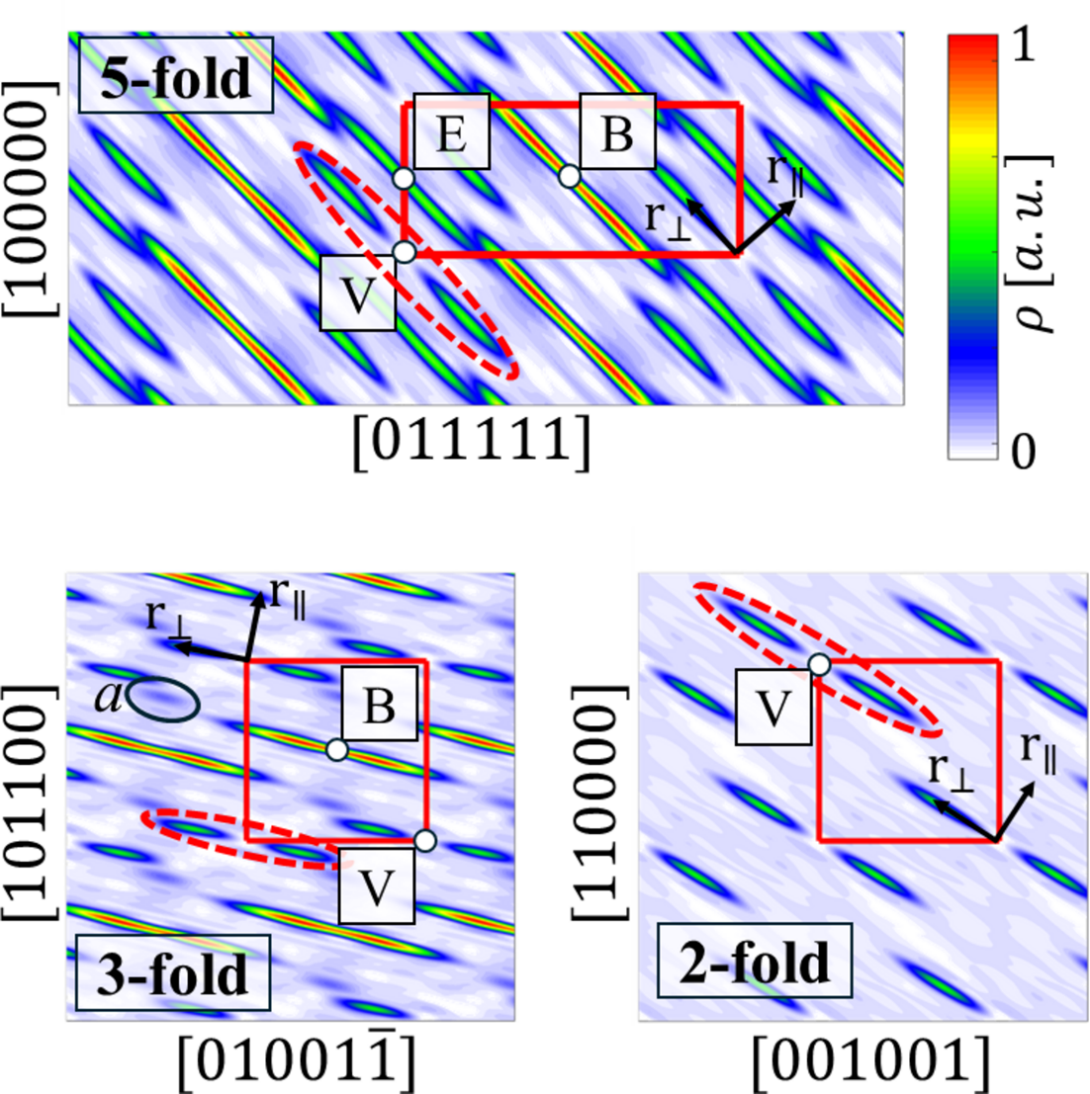
Two-dimensional sections through the 6D electron-density space. Sections involve fivefold, threefold and twofold axes. The 6D unit cell is drawn with a red rectangle. An additional smeared domain in the threefold section is encircled with a black line.

Having obtained the phases of the diffraction peak amplitudes, we are able to calculate the inverse Fourier transform in order to make an initial assessment of the atomic structure. First, we analyse the 6D space by introducing 2D sections in planes of high-symmetry axes (Fig. 4 ▸).

Although phase retrieval did not result in a low *R* factor, the domain structure is clearly visible. Sections through the 6D electron density indicate the existence of three occupation domains (ODs): vertex-centred (V), body-centred (B) and edge-centred (E). This is standard in icosahedral quasicrystals [see *e.g.* Yamada *et al.* (2016*a* ▸) and Yamada *et al.* (2017 ▸)]. The difference between the Tsai and Bergman phases is that the high electron density is accumulated in the B-OD of the Bergman phases, whereas heavy elements in the Tsai phases are in the V-OD without empty space in the centre. The obtained OD structure indicates the Bergman-type phase, similar to P-ZnMgTm (Buganski *et al.*, 2020 ▸). The characteristic overlay between E-OD and B-OD is a frequent feature and indicates the phason flip site [see *e.g.* de Boissieu (2008 ▸) and de Boissieu (2012 ▸)]. The overlay in the fivefold 2D section (red dashed line in Fig. 4 ▸) indicates an elongation of atomic electron density along the fivefold symmetry axis which is a result of local atomic flips. There is an additional smeared domain in the threefold section resulting from a split of the V-OD (labelled domain *a*). The same was observed in P-type ZnMgTm (Buganski *et al.*, 2020 ▸) although the domain is more localized here. A similar intensity domain was observed in the Tsai-type phases of Cd–Mg–Yb (Yamada *et al.*, 2017 ▸) analysed for different contents of Mg. The domain was related to the local chemical disorder related to the Cd/Mg ratio. The higher the Mg content, the less intense this additional domain was. Based on that, we can expect positional disorder in a threefold direction.

**Figure 5:**
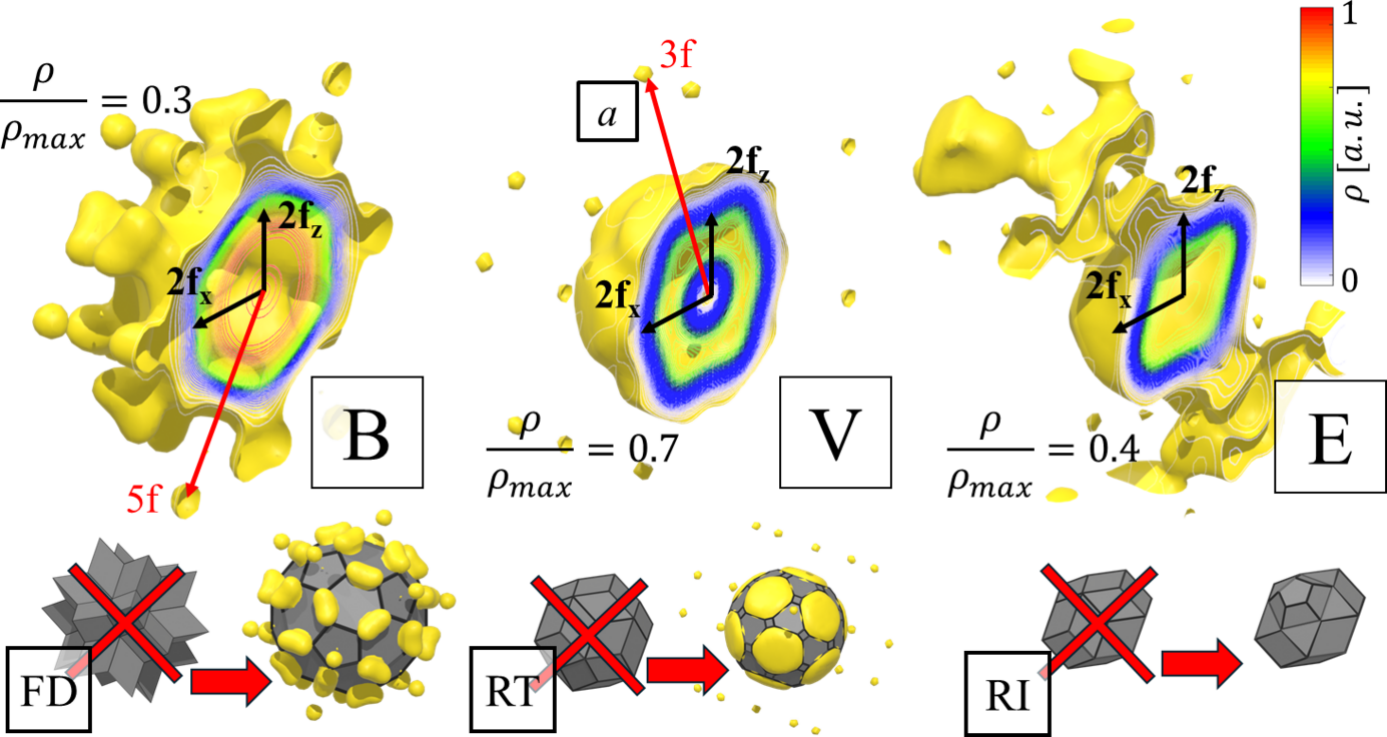
A 3D reconstruction of three ODs calculated at the origin of each OD. A contour plot is placed in the centre of each mid-cut OD. For reference, the shape of the OD in the SD model is shown but, due to mismatch with the isosurface shape, new shapes are being proposed. The cutoff value for each isosurface plot is shown with respect to the maximum electron density (ρ_max_).

Focused analysis can be made when the full 3D reconstruction of the OD is made. Geometric features of ODs are lost when only one dimension of perpendicular space is included. For this purpose, we calculate the 3D inverse Fourier transform in the perpendicular space with real-space coordinates set at the origin of the ODs. The resulting isosurface and contour plots are presented in Fig. 5 ▸. The most important observation is that the shapes of the ODs do not resemble those obtained for the simple decoration model (SD) as introduced for the atomic structure refinement of P-type AlZnMg quasicrystal by Henley & Elser (1986 ▸) and later for P-type AlCuLi by Elswijk *et al.* (1988 ▸). The SD assumes that the atoms occupy vertices, mid-edge positions for both rhombohedra and two additional atoms on the long-body diagonal of the acute rhombohedron. The corresponding ODs in perpendicular space are, respectively, a stellated rhombic triacontahedron or rhombic hexecontahedron [also colloquially called a flower dodecahedron (FD)], an RT and a rhombic icosahedron (RI). In the present case, when the isosurface plot is overlapped with the idealized ODs of the SD model, significant discrepancies are visible. For the B-OD the best fit is given by the truncated icosahedron, specifically the soccerball polyhedron (SB). Athough additional thorns at the vertices of the fitted SB can be seen, the areas of the pentagonal and hexagonal faces are similar to those of the isosurface plot. The shape of the V-OD is well represented by an icosahedron with vertices and edges truncated. It is similar to the B-OD but the additional truncation of edges to the SB was needed to fit the area better. In the case of the E-OD the shape is rather well represented by an RI, but we see an elongation along the fivefold (5f) axis that could be modelled by truncating the top vertex and extruding the pentagonal face along the 5f direction. This face matches the pentagonal face of the B-OD, which is required because the B-OD and E-OD overlap due to the closeness condition [see *e.g.* Quiquandon & Gratias (2014 ▸) and Katz & Gratias (1993 ▸)]. It is therefore evident that the SD model is not a good starting point for the atomic model.

Additional features in the 3D isosurfaces are small domains visible for the V-OD and B-OD. Small domains along the 5f axis for the B-OD are just markers of the E-OD being developed because of their closeness. In contrast, small spherical domains on the threefold (3f) axes around the V-OD are a signature of domain *a* indicated in the 2D section plots in Fig. 5 ▸. The isosurface for the E-OD might look unusual, but additional structures are caused by the B-OD being developed. It forms a much larger structure here than the E-OD in the B-OD plot because it is difficult to find a good cutoff value for the isosurface plot to remove markers of heavy atoms. Heavy atoms show as large electron density, and since the E-OD is occupied by light elements, at any cutoff the B-OD will be visible.

Compared with other known sections of ODs, these sections resemble those for AlCuLi (de Boissieu *et al.*, 1991 ▸). The location of Li agrees with the distribution of Er in our case. The cross section perpendicular to the fivefold axis of the E-OD is more circular for AlCuLi, whereas we see a clear indication of pentagonal structure in the data for our sample. This might be related to poorer statistics of the diffraction peaks used for the AlCuLi structure model.

Electron-density maps for the 3D OD reconstructions can be downloaded as .xsf files from the supporting information.

### 3.2. Physical space analysis

**Figure 6:**
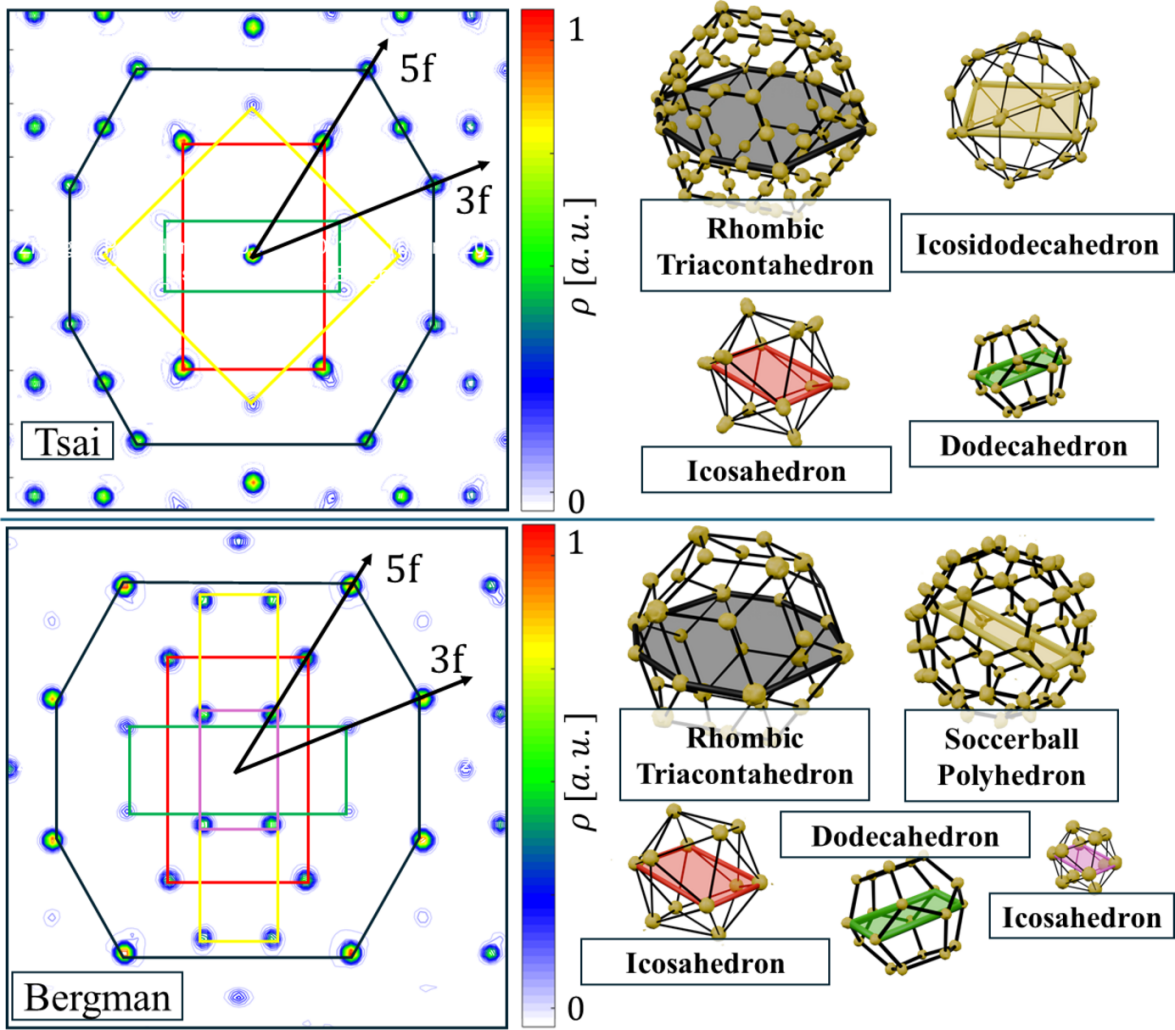
Tsai and Bergman clusters found in 3D real-space electron density. The shell structure is plotted on isosurfaces collected for a threshold value of (1/30) × ρ_max_.

Having analysed the higher-dimensional features of the electron density, it is also useful to examine the real-space structure. Fig. 6 ▸ shows two representative clusters extracted from the 3D real-space electron-density map. Both Tsai and Bergman clusters with complete shell structures can be identified. More disordered variants, with missing atoms, also appear, highlighting the structural complexity. The Tsai cluster contains a central heavy atom, consistent with similar clusters observed in Au–Si–RE approximants, where they strongly influence the magnetic properties (Gebresenbut *et al.*, 2022 ▸). The icosahedral shell displays electron-density elongation along the fivefold axis. No Tsai cluster with a marker of a disordered tetrahedral central unit was found.

Bergman clusters occur in two forms, with and without a central atom. The version lacking a central atom (shown in Fig. 6 ▸) exhibits the best positional order. Although Cd_5.7_Yb is classified as a Tsai-type phase and the model of Takakura *et al.* (2007 ▸) describes it in terms of Tsai clusters, the structure can also be interpreted as a network of interpenetrating Bergman clusters, albeit with positional and chemical disorder (Buganski *et al.*, 2024*a* ▸). The presence of Tsai clusters in the nominally Bergman-type ZnMgEr phase suggests that the structural similarity between the two cluster families is mutual. Studies of various cubic approximants indicate that the main distinctions between Bergman- and Tsai-type phases arise from positional deviations from ideal shell vertices and from partial chemical disorder within the shells (Buganski & Wolny, 2023 ▸).

Although our model does not assume the existence of icosahedral clusters, since it is based directly on the decoration of rhombohedra, it is nonetheless interesting to consider whether a cluster-based model could be constructed. An attempt to build a Bergman-cluster-based model was undertaken by Takakura & Yamamoto (2007 ▸) but it failed to reproduce the experimental data satisfactorily. A likely reason is that, although Bergman-like shell geometry is present in Zn–Mg–RE quasicrystals, the cluster shells are chemically and positionally disordered, preventing the construction of a purely cluster-based structural model.

## 4. Structure refinement

The atomic model of the icosahedral quasicrystal is based on AKNT (Kramer & Neri, 1984 ▸) composed of two rhombohedra. We use the τ^3^ inflated version with an edge length *a* = τ^3^*a*_ico_ ≃ 21.63 Å. The τ^3^ inflation rule for a P-type icosahedral quasicrystal is not rigorously proven (Ogawa, 1985 ▸) but work towards it, *e.g.* using auxiliary dualized AKNT, continues (Fujita, 2023 ▸). The application of AKNT to atomic structure modelling of icosahedral quasicrystals based on a tiling-and-decoration scheme was done in the past for multiple systems, mostly with the SD model [see *e.g.* Henley & Elser (1986 ▸) and Elswijk *et al.* (1988 ▸)]. An alternative approach was designed by Audier & Guyot (1986 ▸, 1988 ▸) who decided to use τ^3^ inflated rhombohedra decorated with two clusters: the Bergman cluster with all the shells, and a τ times smaller RT cluster composed of the first three shells of the Bergman cluster. This approach was used for an Al_57_Cu_10.8_Li_32_ quasicrystal (Guyot & Audier, 2014 ▸; Guyot *et al.*, 1990 ▸). To fill the vast space between the proposed clusters, a pair-distribution function was used. The model was at that time successful, with an *R* factor of 0.16 for 56 X-ray diffraction peaks.

Our approach does not make assumptions about the existence of any cluster, neither Bergman nor Tsai. We turn directly to phased diffraction data and, on that basis, we design the starting model. Even though we have tried to comment on the existence of clusters in the structure, their emergence should be the result of our model and not its primary supposition. What we assume is that AKNT with rhombohedral units is a proper tiling to model the long-range order.

### 4.1. Initial model and refinement strategy

**Figure 7:**
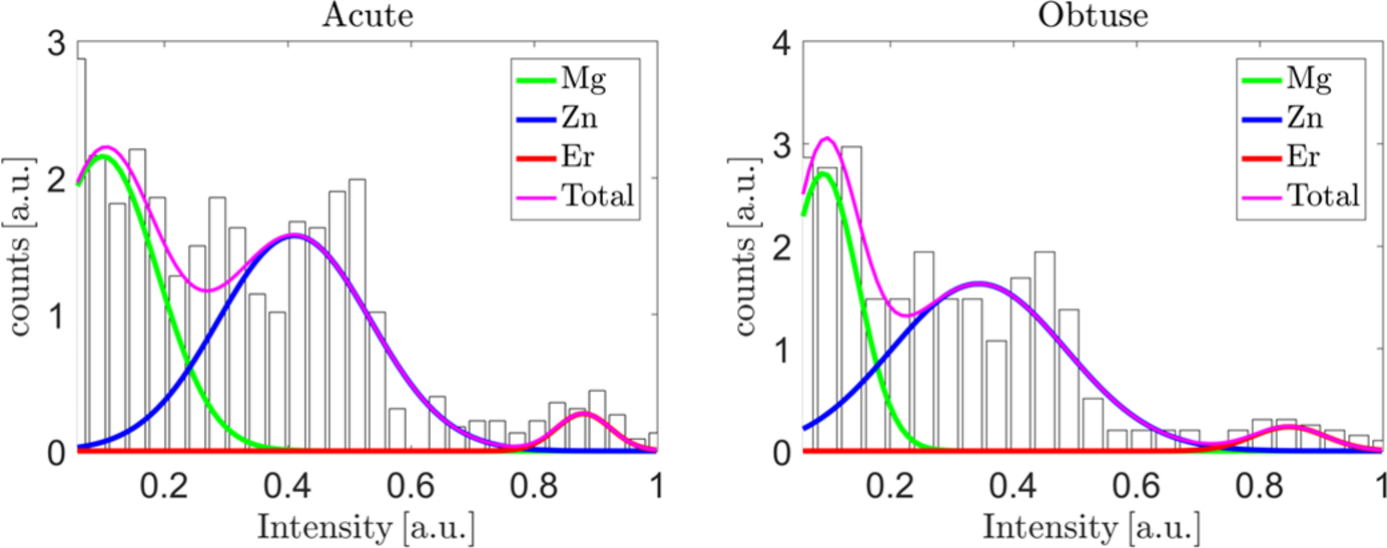
The electron-density map intensity profiles for (left) the acute rhombohedron and (right) the obtuse rhombohedron, with three fitted Gaussian curves corresponding to each atomic type.

The initial model was made by superimposing AKNT on the electron-density map and assigning atoms to local maxima. The initial atomic decoration is based on the intensity of the electron-density profile (Fig. 7 ▸). Three Gaussian curves are fitted to the intensity profile, each corresponding to one atomic type. For the overlapped part of the fitted functions, the mixed occupancy is set for further refinement. As part of the initial model, *m*3 point symmetry is applied to each rhombohedron to reconstruct full icosahedral symmetry. The fivefold symmetry is encoded in the fact that each rhombohedron occurs in ten orientations. The mathematical model used for the calculation of the structure factor is based on a tiling-and-decoration scheme (Strzalka & Wolny, 2014 ▸; Strzalka *et al.*, 2015 ▸) with the average unit-cell approach for the distribution of tile reference vertices [see *e.g.* Wolny *et al.* (2016 ▸) and Strzalka *et al.* (2016 ▸)]. The list of refined atomic positions is given in Table S1.

The model involves 344 independent parameters, which is a significant simplification compared with the P-type ZnMgTm model (>700 parameters) and the P-type CdYb model (>400 parameters) (Buganski *et al.*, 2024*a* ▸). Only isotropic phononic atomic displacement parameters are used. The phason correction is utilized with a Gaussian term (Bancel, 1989 ▸; Lubensky *et al.*, 1986 ▸). One extinction parameter is used. The number of diffraction peaks used for structural refinement is the same as that for the *ab initio* structure solution, which makes the peak-to-parameter ratio equal to 7.77. The strategy for the refinement involved sequentially conducting refinement of atomic positions and phonons, with complete refinement of the parameters at the end. At each stage, we do approximately 50 iterations of a gradient-descent algorithm. Optimization of the phasonic atomic displacement parameter was always activated.

### 4.2. Model evaluation against X-ray diffraction data

The final value of the crystallographic *R* factor is 0.1407 and the weighted *R* factor is around 0.025. The refined composition is Zn_69.162_Mg_21.271_Er_9.567_ with a 0.0633 Å^−3^ point density. The refined composition with respect to the experimental one deviates only within 1 at.%, which is a very good result for a ternary phase. The point density is almost identical to P-type ZnMgTm (Buganski *et al.*, 2020 ▸).

The refined value of the phasonic *B* factor is 0.23 Å^2^, which is almost ten times lower than the value for icosahedral ScZn, which is 2.05 Å^2^ (Yamada *et al.*, 2016*a* ▸). However, the phasonic *B* factor obtained from diffuse scattering data of ScZn is 0.76 Å^2^, indicating that the atomic model used for ScZn overestimates phasons. Compared with other Bergman-type quasicrystals, the value refined here is greater than for Zn–Mg–Tm where it could even be set to 0 without impacting the *R* factor. In this case, setting the phasonic *B* factor to 0 results in an *R* factor of 0.19. We believe that a higher value of the phasonic parameter is a result of worsening data quality, which will be further proven in the context of phonons. However, the use of AKNT consistently shows a lower phasonic *B* factor than models based on atomic clusters embedded in 6D space. Comparison with diffuse scattering done for ScZn indicates that the cluster model is too idealistic, resulting in a higher phasonic *B* factor.

**Figure 8:**
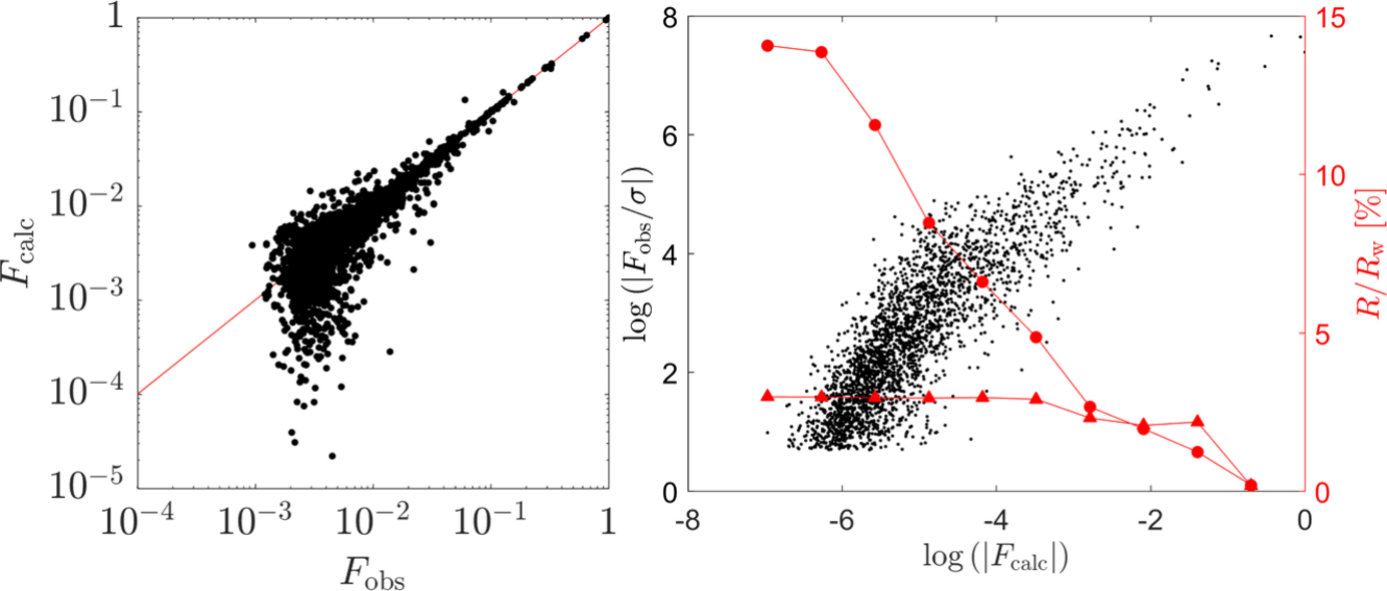
(Left) Correlation plot between the calculated and observed diffraction peak amplitudes. (Right) Distribution of the normalized observed diffraction amplitudes with respect to the calculated values (black). As indicated by red curves, the crystallographic *R* factor grows with the inclusion of small magnitude peaks (circles), but the weighted *R*_w_ is constant in value (triangles). This indicates that the intensity of the small peaks cannot be accurately estimated.

In Fig. 8 ▸(left), we show a correlation plot between the calculated and experimental values of the diffraction amplitudes. The plot is linear in the high magnitude part but has a characteristic bias in the low-magnitude region that is attributed to both the phason disorder and the multiple scattering mechanism (Buganski *et al.*, 2016 ▸; Buganski *et al.*, 2019 ▸; Fan *et al.*, 2011 ▸). In Fig. 8 ▸(right), the distribution of peak intensities related to their standard deviation is shown. The data are significantly scattered even for high-intensity peaks. This means that the data quality certainly affects the accuracy of the model. The low value of *R*_w_, plotted with triangles, is proof of the dominant impact of the uncertainties in the peak intensities. As observed for quasicrystals, the value of the *R* factor depends on the magnitude of the peaks considered in the calculations. The lowest value is achieved for the highest magnitude peaks, with a steep upward slope below 10^−2^ peaks. In contradiction, *R*_w_ grows slowly up to 10^−3^ and saturates.

### 4.3. Atomic model

**Figure 9:**
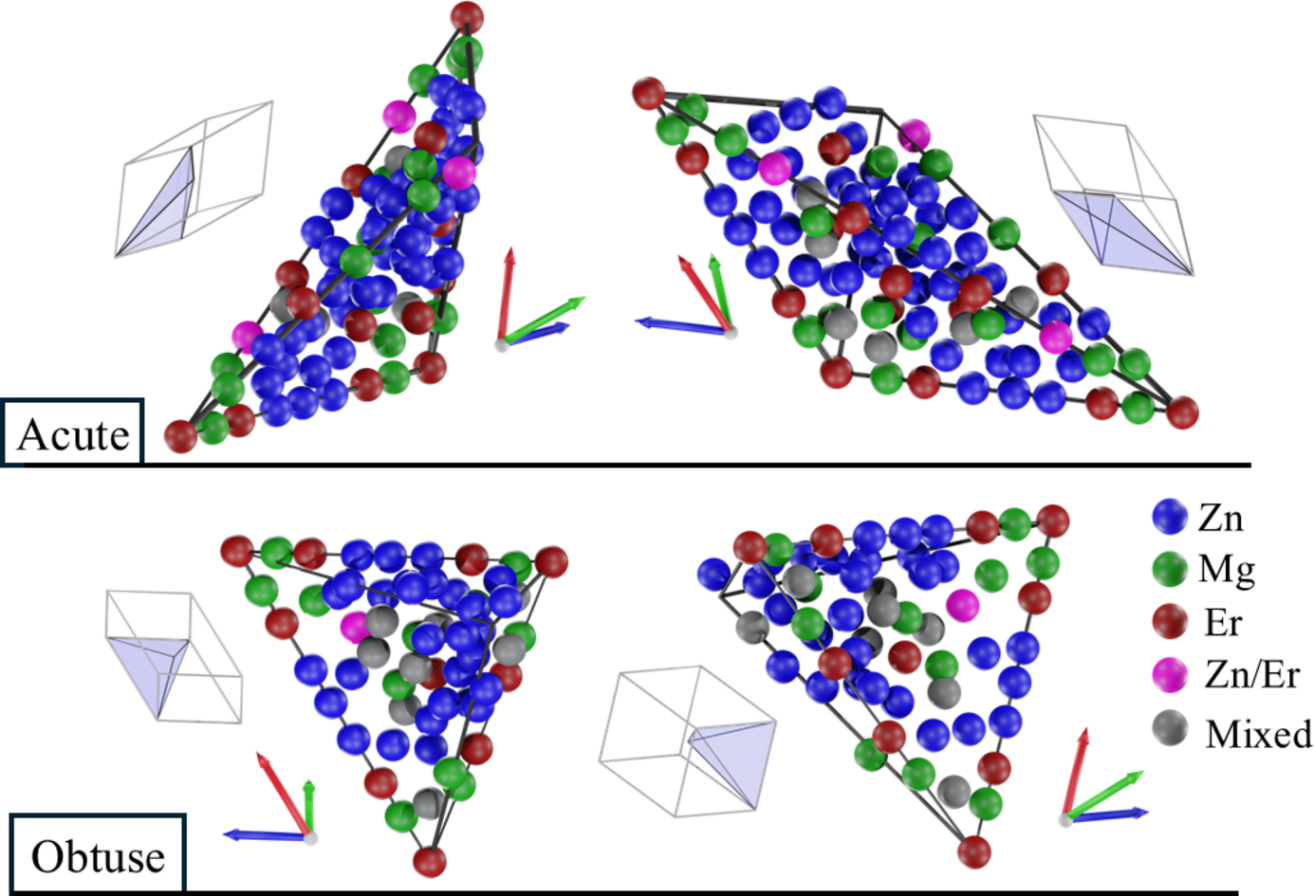
Asymmetric parts of the rhombohedra of AKNT with atomic decoration in P-type ZnMgEr. The orientation of each unit is referred to the orientation within the fully reconstructed rhombohedron.

The number of atoms in the asymmetric part of the acute rhombohedron is 87 and for the obtuse it is 59. Of these, nine are mixed occupancy sites for both rhombohedra. On the mixed site, occupancy by Er is low, being around 18% at most, with no mixed Mg/Er site. Better quality data are required to detect a mixed occupancy site of Mg/Er because of a high X-ray radiation attenuation difference. Among the mixed Zn/Mg sites, the occupancy is almost even for both elements. The atomic decoration of the asymmetric parts of the rhombo­hedral units is shown in Fig. 9 ▸. Because of the potential interest in Er sites due to their impact on magnetic properties, Zn/Er sites are plotted separately. The orientation of the units is indicated by three vectors of an icosahedral setting where the vectors point from the centre toward the common face of an icosahedron. Each unit is shown in two orientations, with the orientation of the asymmetric unit given in the context of the full rhombohedral unit.

Atomic positions in the asymmetric units are available as a .cif file in the supporting information but, due to the fact that the structure is aperiodic, the space symmetry was set to *P*1 in the file. The list of atomic species is also provided as a table in the supporting information (Table S1). The atomic structure can be visualized with .blend files that can be opened with *Blender* (Version 4.4 or higher; https://www.blender.org/). These files are deposited in the Open Science Framework (https://osf.io/8r5bd).

**Figure 10:**
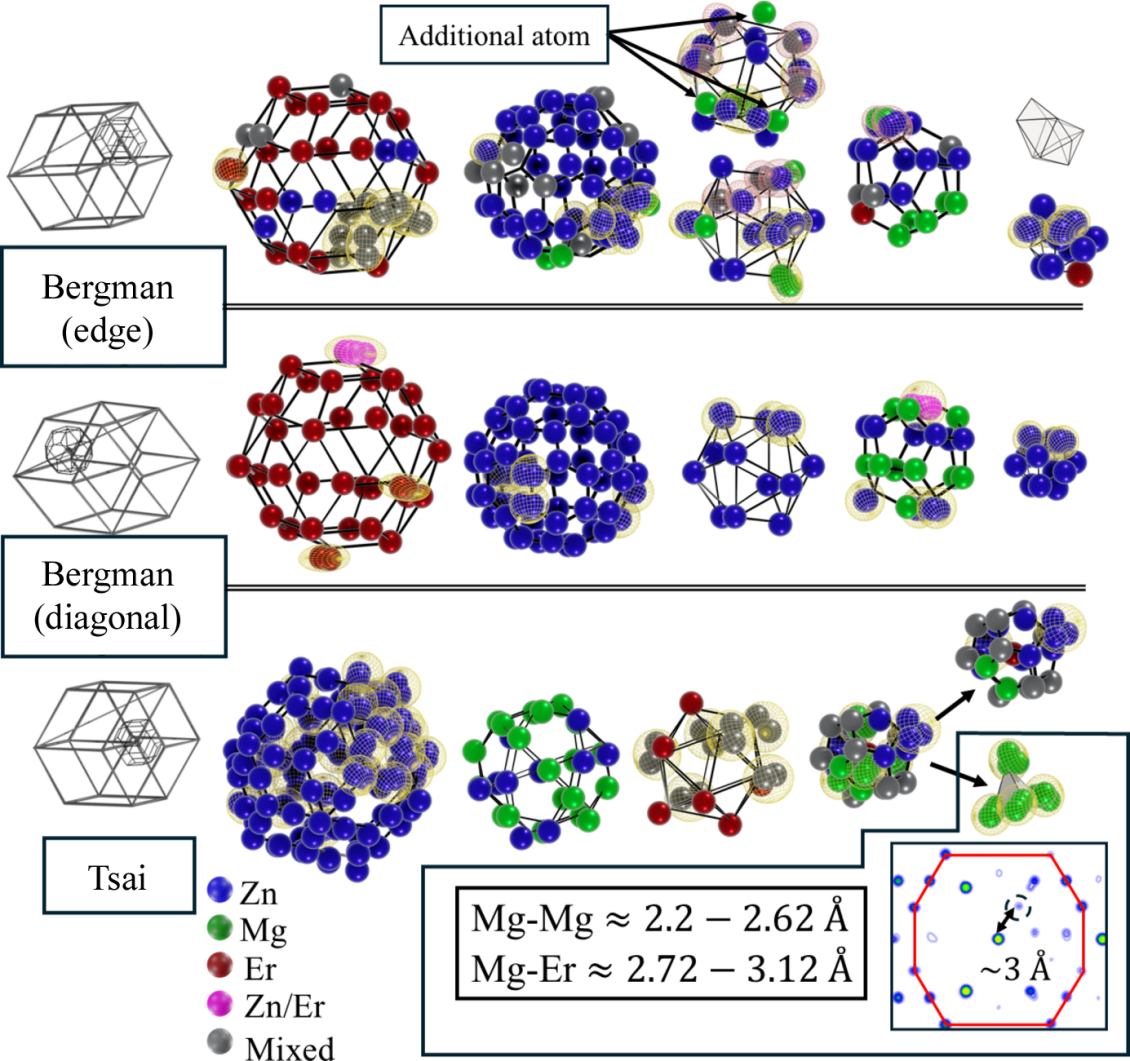
The local atomic environment is based on clusters, with the last shell being an RT polyhedron found in the rhombic dodecahedron composed of two acute rhombohedra and two obtuse rhombohedra. Two types of atomic clusters, Bergman and Tsai, are studied as examples to reveal structural properties. Short Mg–Mg bonds are observed in the dodecahedral shell of the Tsai cluster, created by the Mg4 tetrahedron. The observation of additional atoms in the dodecahedral shell is confirmed with the electron-density map. Split atoms due to deviation from high-symmetry Wyckoff sites are shown with a wireframe mesh for clarity of presentation.

More detailed structural features can be analysed by examining the local assembly of the structural units. Here, the key unit is a rhombic dodecahedron composed of two acute and two obtuse rhombohedra (Fig. 10 ▸). Within this unit, we identify atomic clusters whose outer shells are RT. Cluster-based descriptions have historically been successful for icosahedral quasicrystals, particularly for Tsai-type (Takakura *et al.*, 2007 ▸; Yamada *et al.*, 2016*b* ▸) and Mackay-type structures (Yamada *et al.*, 2024 ▸), but are less useful for decagonal phases (Steurer, 2006 ▸) where they seem to provide a geometric framework. Whether clusters truly act as structure-stabilizing units remains unresolved. For Al-based Mackay phases, inter-cluster bonding has been demonstrated (Iwasaki *et al.*, 2023 ▸), offering evidence for electronic stabilization, but similar confirmation is lacking for other families. Whether clusters play a stabilizing role in Bergman-type quasicrystals, despite their prominence in periodic approximants such as 2/1 AlZnMg (Lin & Corbett, 2006 ▸) and AlCuLi (Guyot & Audier, 2014 ▸), is still uncertain. Our refined model provides insight into this question.

One of the RT clusters is centred along the long body diagonal of the acute rhombohedron. This cluster closely matches the ideal Bergman shell geometry, showing only minor displacements from the expected shell vertices. Some atomic sites appear split due to shifts from Wyckoff positions along the threefold axis of the rhombohedron. The RT shell is fully occupied by Er atoms, except for a single mixed Zn/Er site (82%/18%). Apart from the dodecahedral shell, all shells exhibit monatomic decoration. The shortest Mg–Mg distance within the dodecahedron is 3.04 Å, typical for Zn–Mg alloys. This cluster represents a textbook Bergman configuration.

A second Bergman-type RT cluster is located at the edge of an acute rhombohedron shared with two obtuse rhombohedra in the rhombic do­deca­hedron. This cluster is significantly more chemically disordered, with no shell containing a single element. Its innermost icosahedron contains several vacant vertices due to proximity to Mg atoms in adjacent dodecahedral and icosahedral shells. Notably, additional Mg atoms occur above the mid-edge positions of the icosahedral shell (third shell from the centre). This feature has been observed in many Tsai-type approximants, for example 1/1 CdYb (Buganski & Wolny, 2023 ▸), and was examined in electronic stabilization studies of 1/1 Zn–Mg–Sc (Buganski *et al.*, 2024*b* ▸) and 1/1 Zn–Mg–Hf (Buganski *et al.*, 2025 ▸). The first two systems are Tsai-type phases, while the last contains a pseudo-Bergman cluster in which deviations from the ideal structure arise from the possible presence of an octahedral inner shell and Mg-deficient occupancy in the dodecahedron (Fujita *et al.*, 2024 ▸). The appearance of an additional atom in the icosahedral shell and the violation of standard shell chemistry in ZnMgEr strongly suggest that so-called Bergman-type quasicrystals cannot be represented solely by ideal Bergman clusters. This observation challenges the applicability of cluster-based descriptions for this family and may explain why generalizing the OD-based approach used successfully for icosahedral CdYb (Takakura *et al.*, 2007 ▸) led to poor agreement for ZnMgHo (Takakura & Yamamoto, 2007 ▸).

A representative Tsai cluster appears at the vertex inside the rhombic dodecahedron, a position shared by all constituent rhombohedra. Variants of the Tsai cluster are found at all rhombohedral vertices and ultimately their shell structure will depend on the rhombohedra vertex environment. In this environment, the Tsai cluster is chemically and positionally disordered relative to the ideal structure. A key distinguishing feature is the presence of additional atoms in the inner dodecahedron: four Mg atoms located near the centres of pentagonal faces, forming a tetrahedral Mg_4_ unit. The distance between one of these Mg atoms and the nearest dodecahedral vertex Mg atom is approximately 2.2 Å. Although unusually short, this distance is physically plausible for complex intermetallics. In the ZnMg(Hf, Zr, Ti) 1/1 approximant (Gómez *et al.*, 2008 ▸), the Zn10/Mg10–Mg2 distance is ∼2.8 Å, while the Zn8/Mg8–Zn14 distance is ∼2.2 Å. In ZnMgTi, the Zn14 site is even Mg-occupied, producing a similar short bond. Density functional theory calculations on Zn–Mg–Hf (Buganski *et al.*, 2025 ▸) also predict short Mg–Mg separations associated with enhanced electron localization and multi-centre bonding with neighbouring Zn atoms. Given the structural similarity between approximants and quasicrystals, such short Mg–Mg distances can be therefore expected in P-ZnMgEr. The Mg–Er distances are likewise short (2.6–3.1 Å), indicating local accumulation of electropositive species. The additional atom in the dodecahedral shell is also visible in the real-space electron-density map (Fig. 10 ▸). The separation between this site and the central atom matches the refined value, and missing positions in this shell correspond to low electron density, consistent with the model. No anomalous bond distances occur in other Tsai-cluster shells, although some sites are split.

The Mg_4_ tetrahedron in the Tsai-type cluster could alternatively arise from misassigning electron density to Mg_4_ rather than to a partially occupied Zn_4_ tetrahedron that shares occupancy with the central Er. Because Mg scatters weakly and Zn may appear with fractional occupancy, distinguishing these possibilities is beyond the resolution of the present data. It is unclear whether even high-quality synchrotron X-ray diffraction would fully resolve this ambiguity. Tetrahedral inner shells are common in Tsai-type quasicrystals [see *e.g.* Euchner *et al.* (2013 ▸) and Ishii *et al.* (2025 ▸)], but their occurrence in Bergman-type systems warrants further investigation.

In summary, we have presented here a local structural analysis based on several representative clusters. Many more clusters exist in the structure, consistent with the previously established covering by RT clusters connected through short *a* linkages in the isostructural ZnMgTm phase (Buganski *et al.*, 2020 ▸). Although analysing all clusters is impractical, a clear pattern emerges. Well defined Bergman clusters appear along the long body diagonal of the acute rhombohedron, but most clusters deviate from the ideal geometry and should be regarded as pseudo-Bergman clusters. Similarly, Tsai clusters, or more accurately pseudo-Tsai clusters, also occur. In our refined model, the cluster shells show no systematic chemical ordering that would support constructing the entire structure from ideal chemically pure Bergman clusters.

### 4.4. Phonons

**Figure 11:**
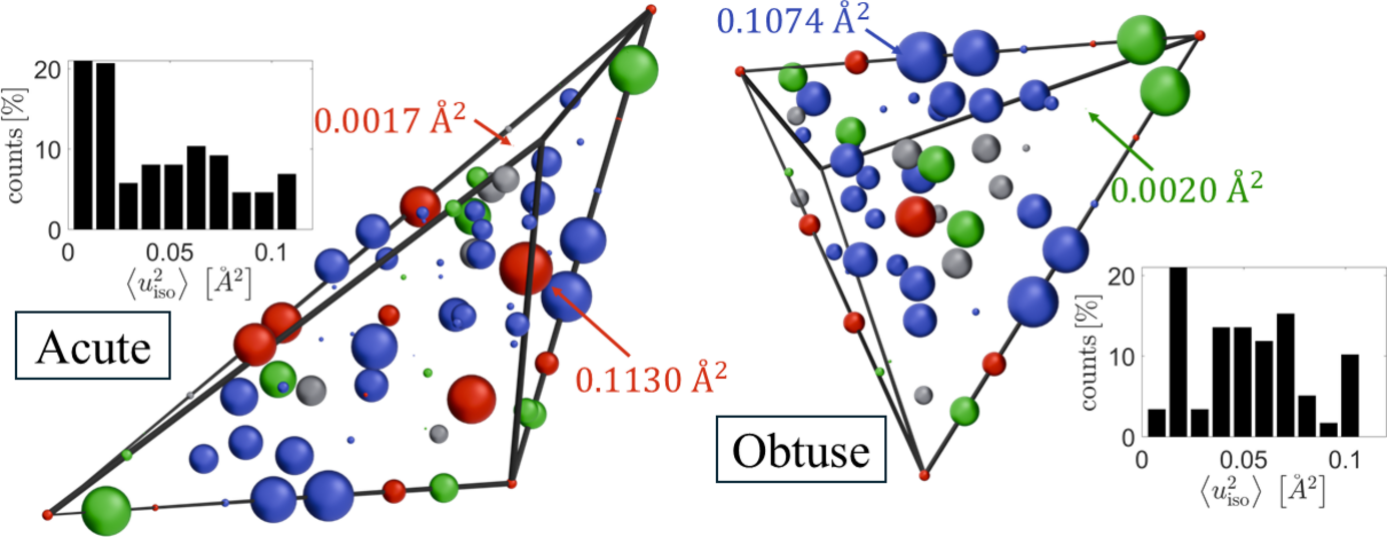
Refined values of phononic *B* factors plotted for the corresponding atoms in asymmetric parts of rhombohedral units. The radius of a sphere is proportional to the *B* factor value. The inset histograms show the distribution of the mean atomic displacement in both units. Colours as in Fig. 9 ▸.

The parameters related to phonons give a better understanding of the quality of the structural refinement. Abnormally high values of phononic *B* factors modelled with the Debye–Waller correction may indicate a split atomic site or too high a local atomic density. In our case, the quality of the X-ray diffraction data impacts high values of the refined phononic mean displacement parameters. In Fig. 11 ▸, the values of the phononic *B* factors are plotted for both asymmetric parts of the rhombohedral units used for the structural refinement. The radius of a sphere is proportional to the *B* factor and the colours indicate the type of atom, as in Fig. 9 ▸. In the inset histograms, the distribution of isotropic mean atomic displacement, 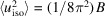, is shown. No correlation can be detected between the type, position and *B* factor value. Small (<0.01 Å^2^) and large (>0.07 Å^2^) *u*_iso_ occur for constrained (vertex, edge and face bound) and unconstrained atoms. Compared with *u*_iso_ found in the Zn–Mg–(Hf, Zr, Ti) 1/1 approximant phase, the values here are high (Gómez *et al.*, 2008 ▸). The highest value for Zn–Mg–Hf/Zr, equal to 0.120/0.173 Å^2^, occurs for Zn18 – an atom found in the disordered dodecahedron of the cluster at the cube body-centre position. In our case, the highest value is 0.1130 Å^2^ and concerns an Er atom from one of the rhombic triacontahedral outer shells in the acute rhombohedron. The important difference is that our data were collected at 100 K, while the data for Zn–Mg–(Hf, Zr, Ti) were collected at ambient temperature. We are able to compare our result directly with refined phonons for another Bergman-type quasicrystal, Zn–Mg–Tm (Buganski *et al.*, 2020 ▸). In that case as well, the presently refined values are high, considering 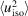 for Zn–Mg–Tm are all below 0.07 Å^2^ and the data were also collected at ambient temperature. Higher values of phonon-related parameters are related to worse data quality, evident from the high *R*_int_ value and the low number of symmetry-independent parameters limited to 1σ peaks.

### 4.5. Residual electron density

**Figure 12:**
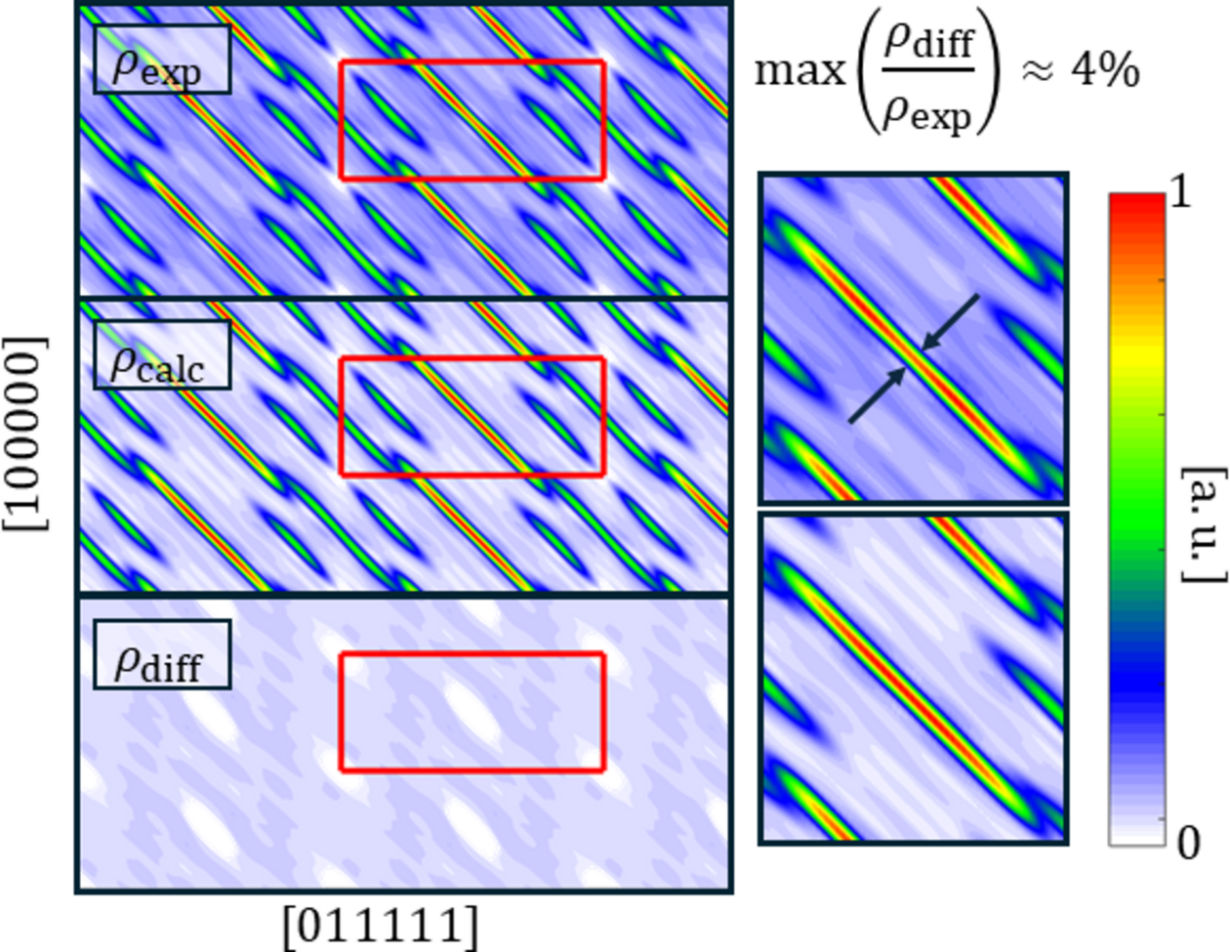
Maps of the experimental electron density (ρ_exp_), the electron density calculated based on the model (ρ_calc_) and the difference (ρ_diff_) in the plane of two perpendicular fivefold symmetry axes. The excess in the calculated density of the B-OD is visible.

The residual electron density is below 4% of the experimental value derived from the measured diffraction amplitudes. Fig. 12 ▸ shows the experimental, model-calculated and difference electron-density maps for a 2D section along two fivefold symmetry axes of the 6D unit cell. All three ODs are visible in this section. The most significant discrepancy appears in the B-OD, where heavy elements accumulate. The calculated density shows an excess of intensity at the centre of the B-OD, indicating that the model may contain too many heavy atoms in this region. This interpretation is consistent with the slight difference between the experimental Er content (8.9 at.%) and the refined value (9.567 at.%). With the current data quality, this discrepancy cannot be resolved. The most plausible explanation is that the atomic site with the highest phononic mean displacement parameter (Fig. 11 ▸) should be treated as a mixed-occupancy site. Another possibility is unaccounted mixing between Er and the Mg_4_ tetrahedron at the centres of Tsai clusters located at rhombohedral vertices. Higher-resolution data will be required to distinguish between these scenarios.

### 4.6. Structure in six dimensions

Our structure model is constructed entirely in 3D space using the tiling-and-decoration scheme. We did not employ the higher-dimensional approach in which the ODs are explicitly defined – typically through cluster-based decoration in 6D crystallography [see *e.g.* Takakura *et al.* (2007 ▸)]. However, because many researchers in quasicrystal crystallography benefit from a 6D representation, we reconstructed the ODs corresponding to our 3D model.

Determining the ODs from a 3D refinement is nontrivial, as the assignment of atoms to individual domains is not unique. We followed the procedure used for ZnMgTm (Buganski *et al.*, 2020 ▸) and AlCuRh (Kuczera *et al.*, 2012 ▸). A large approximant-like slab containing roughly one million atoms was generated. For each atom, a 6D vector was constructed by taking the refined 3D coordinates and setting the perpendicular-space components to zero. This vector was multiplied by the inverse of the 6×6 projection matrix (Yamamoto, 1996 ▸) to obtain reduced 6D coordinates. These coordinates were wrapped into a single 6D unit cell by restricting all components to the interval [0, 1].

**Figure 13:**
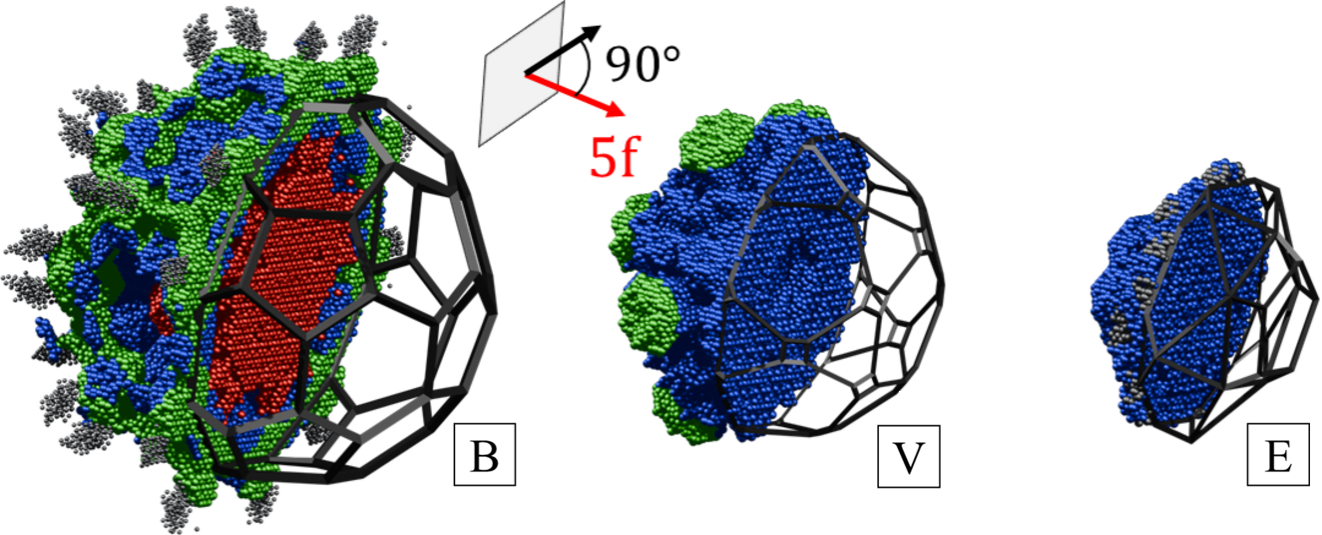
ODs resulting from lifting the 3D atomic model to a 6D higher-dimensional space. Colours are same as for the atoms in Fig. 9. Polyhedra best fitted to the isosurface plots in Fig. 5 are also used here for reference.

To assign each lifted atom to one of the three ODs, we calculated the distance between its real-space position and the reference ODs. If this distance was less than 0.5 Å, the atom was attributed to that OD, and its perpendicular-space co­ordinates were recovered relative to the OD’s centre. The resulting lifted atoms are shown in Fig. 13 ▸, where each OD is cut by a plane perpendicular to a fivefold symmetry axis. The reconstructed OD geometries agree well with those obtained directly from the 3D electron-density isosurfaces (Fig. 5 ▸). For comparison, idealized OD prototypes are included, illustrating clear deviations from the SD model.

**Figure 14:**
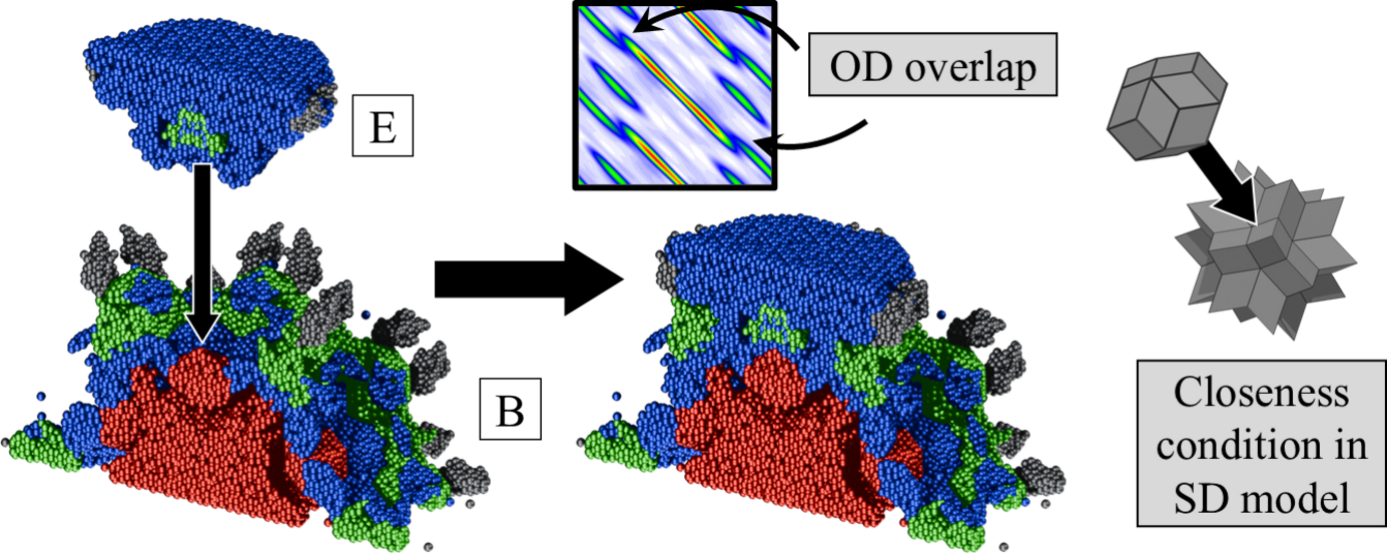
The closeness condition shown based on the overlap of the reconstructed B-OD and E-OD. The same is shown on the far right for ODs in the SD model for reference.

The reconstructed ODs confirm that Er occupies the centre of the B-OD, while the V-OD exhibits an empty centre. Mg and Zn are distributed across all domains, with Mg in the E-OD clearly visible in Fig. 14 ▸. The overall OD shapes resemble those found for ZnMgTm, but with notable improvements. In ZnMgTm, the ODs were interpreted using the SD model because alternative shapes were not considered. The present ODs show better agreement with both the refined atomic positions and the phased electron-density data. These newly established OD shapes are also compatible with ZnMgTm when differences in resolution are taken into account. In ZnMgTm, Mg appeared in the outer layer of the E-OD due to limited resolution in the lifting procedure, causing some atoms to be misassigned to the B-OD because the two domains overlap under the closeness condition. A similar effect resulted in excess atoms near the fivefold faces of the B-OD. In the present reconstruction, a clear hollow region is observed where the E-OD fits into the B-OD (Fig. 14 ▸). In ZnMgTm this hollow was absent because atoms belonging to the E-OD were also assigned to the B-OD, effectively duplicating them due to the chosen distance threshold. After correcting for these issues, the ODs of ZnMgTm and ZnMgEr show the same overall topology.

The closeness condition [see *e.g.* Quiquandon & Gratias (2014 ▸) and Katz & Gratias (1993 ▸)] for the reconstructed ODs in ZnMgEr is most evident in the overlap between the B-OD and E-OD, as shown in Fig. 14 ▸. Their overlap in perpendicular space is also visible in the extracted 2D section of the electron-density map. Along the fivefold axis, the E-OD fits into the hollow region of the B-OD, where the surrounding Mg atoms and mixed-occupancy sites can be assigned to either OD with comparable accuracy. The Mg atoms located in the centre of the E-OD are also clearly visible here, an observation that was not possible in the section shown in Fig. 13 ▸.

## 5. Summary

The atomic structure of the primitive icosahedral Zn_70.83_Mg_20.31_Er_8.86_ phase was solved in physical space with a model based on Ammann–Kramer–Neri tiling. For this purpose, a set of in-house X-ray diffraction data was used. The model is characterized by a moderate value of the crystallographic *R* factor of 0.1407 based on 2674 peaks and 344 parameters. This is an updated version of the model used for icosahedral Zn–Mg–Tm with constraints put on atoms located on the vertices, edges and faces of the rhombohedral units.

Analysis of the local atomic structure provides evidence for both Bergman and Tsai clusters, which are present in the electron-density maps based on diffraction peaks phased with the *SUPERFLIP* software and in the refined structure model. Bergman clusters were shown to be represented by a positionally ordered version with a clear distribution of chemical species between shells, and a chemically disordered version with vacancies in the innermost icosahedral shell. The example Tsai cluster is chemically disordered but preserves the geometry of the cluster shells. The Mg4 tetrahedron is found at the centre but it is possible that the proper unit is a mixed occupancy of Zn_4_ and Er at the centre. If Mg_4_ is indeed the real unit, the Mg–Mg distance is short, being around 2.2 Å. Such a short distance could be explained by the formation of multicentre bonds with neighbouring Zn atoms, as shown for the Zn–Mg–Hf cubic phase. The presence of both Bergman and Tsai versions of rhombic triacontahedral clusters agrees with former observations made in icosahedral quasicrystals such as Zn–Mg–Tm or Cd–Yb and also in cubic approximant crystals.

The drawback of the quality of the collected data is evident from the high values of the refined phononic parameters, even though the experiment was carried out at 100 K. However, the refined positions of the atoms and the composition are physically relevant. Observations correlate with previously reported models.

The reconstruction of the occupation domains in perpendicular space allows us to confirm similarity with the ZnMgTm phase model. For both structures, a simple decoration model consisting of the atomic decoration of rhombohedra at vertices and mid-edge positions and along the long body diagonal of the acute rhombohedron is not a good approximation. Modified idealized occupation domains that well approximate the obtained shapes of the occupation domains are presented. These are a truncated icosahedron, an icosahedron with edges and vertices truncated, and a modified rhombic icosahedron with vertices at fivefold axes truncated into pentagonal faces and later extruded. They could potentially be used to construct a 6D model of the structure, but careful analysis of closeness conditions must still be made.

## Supplementary Material

Crystal structure: contains datablock(s) ZnMgEr_Acute_asymmetric, ZnMgEr_Obtuse_asymmetric. DOI: 10.1107/S205252062600003X/ra5158sup1.cif

Structure factors: contains datablock(s) ZnMgEr. DOI: 10.1107/S205252062600003X/ra5158ZnMgErsup2.hkl

List of refined positions and analysis of sample composition. DOI: 10.1107/S205252062600003X/ra5158sup3.pdf

Electron density around B-OD (as .xsf). DOI: 10.1107/S205252062600003X/ra5158sup4.txt

Electron density around V-OD (as .xsf). DOI: 10.1107/S205252062600003X/ra5158sup5.txt

Electron density around E-OD (as .xsf). DOI: 10.1107/S205252062600003X/ra5158sup6.txt

CCDC references: 2520367, 2520368

## Data Availability

Data are available as supporting information. Single-crystal X-ray diffraction data have been deposited in the Open Science Framework (https://osf.io/8r5bd).

## References

[bb1] Agilent (2014). *CrysAlis PRO.* Agilent Technologies Ltd, Yarnton, Oxfordshire, England.

[bb2] Audier, M. & Guyot, P. (1986). *Philos. Mag. B* **53**, L43–L51.

[bb3] Audier, M. & Guyot, P. (1988). *Philos. Mag. Lett.* **58**, 17–23.

[bb4] Bancel, P. A. (1989). *Phys. Rev. Lett.* **63**, 2741–2744. 10.1103/PhysRevLett.63.2741 10040978

[bb5] Buganski, I. & Bindi, L. (2021). *IUCrJ* **8**, 87–101. 10.1107/S2052252520015444 PMC7792992 33520245

[bb6] Buganski, I., Strzalka, R. & Wolny, J. (2016). *Phys. Status Solidi B* **253**, 450–457.

[bb7] Buganski, I., Strzalka, R. & Wolny, J. (2019). *Acta Cryst.* A**75**, 352–361. 10.1107/S2053273318017254 30821268

[bb8] Buganski, I., Strzalka, R. & Wolny, J. (2024*a*). *Acta Cryst.* B**80**, 84–93. 10.1107/S2052520624000763 38441050

[bb9] Buganski, I., Strzalka, R. & Wolny, J. (2024*b*). *Isr J. Chem.* **64**, e202300139.

[bb10] Buganski, I., Vrtnik, S., Strzalka, R., Luzar, J., Wolny, J. & Fujita, N. (2025). *Sci. Rep.* **15**, 28609. 10.1038/s41598-025-13835-1 PMC12325726 40764639

[bb11] Buganski, I. & Wolny, J. (2023). *J. Alloys Compd.* **939**, 168823.

[bb12] Buganski, I., Wolny, J. & Takakura, H. (2020). *Acta Cryst.* A**76**, 180–196. 10.1107/S2053273319017339 PMC7053224 32124856

[bb13] De Boissieu, M. (2008). *Philos. Mag.* **88**, 2295–2309.

[bb14] de Boissieu, M. (2012). *Chem. Soc. Rev.* **41**, 6778–6786.

[bb15] de Boissieu, M., Janot, C., Dubois, J. M., Audier, M. & Dubost, B. (1991). *J. Phys. Condens. Matter* **3**, 1–25.

[bb16] Dolinšek, J., Jagličić, Z., Chernikov, M. A., Fisher, I. R. & Canfield, P. C. (2001). *Phys. Rev.* B**64**, 224209.

[bb17] Elser, V. (1986). *Acta Cryst.* A**42**, 36–43.

[bb18] Elswijk, H. B., De Hosson, J. Th. M., van Smaalen, S. & de Boer, J. L. (1988). *Phys. Rev. B* **38**, 1681–1685. 10.1103/physrevb.38.1681 9946451

[bb19] Euchner, H., Yamada, T., Rols, S., Ishimasa, T., Kaneko, Y., Ollivier, J., Schober, H., Mihalkovič, M. & de Boissieu, M. (2013). *J. Phys. Cond. Matter* **25**, 115405. 10.1088/0953-8984/25/11/115405 23411496

[bb20] Fan, C. Z., Weber, T., Deloudi, S. & Steurer, W. (2011). *Philos. Mag.* **91**, 2528–2535.

[bb21] Fisher, I. R., Cheon, K. O., Panchula, A. F., Canfield, P. C., Chernikov, M., Ott, H. R. & Dennis, K. (1999). *Phys. Rev. B* **59**, 308–321.

[bb22] Fisher, I. R., Islam, Z., Panchula, A. F., Cheon, K. O., Kramer, J., Canfield, P. C. & Goldman, A. I. (1998). *Philos. Mag. B* **77**, 1601–1615.

[bb24] Fujita, N. (2023). *J. Phys. Conf. Ser.* **2461**, 012007.

[bb25] Fujita, N., Mihalkovič, M. & Henley, C. L. (2024). *Isr. J Chem.* **64**, e202300130.

[bb26] Gebresenbut, G. H., Shiino, T., Andersson, M. S., Qureshi, N., Fabelo, O., Beran, P., Qvarngård, D., Henelius, P., Rydh, A., Mathieu, R., Nordblad, P. & Pay Gomez, C. (2022). *Phys. Rev. B* **106**, 184413.

[bb27] Gómez, C. P., Ohhashi, S., Yamamoto, A. & Tsai, A. P. (2008). *Inorg. Chem.* **47**, 8258–8266. 10.1021/ic800874u 18702481

[bb28] Guyot, M., Audier, M. & de Boissieu, M. (1990). *Quasicrystals and Incommensurate Structures in Condensed Matter*, edited by M. J. Yacamán, D. Romeu, V. Castaño & A. Gómez, pp. 251–259. World Scientific.

[bb29] Guyot, P. & Audier, M. (2014). *C. R. Phys.* **15**, 12–17.

[bb30] Hann, C. T., Socolar, J. E. S. & Steinhardt, P. J. (2016). *Phys. Rev. B* **94**, 014113.

[bb31] Henley, C. L. & Elser, V. (1986). *Philos. Mag. B* **53**, L59–L66.

[bb32] Ishii, H., Yoko, K., Kanai, T., Deguchi, K., Labib, F., Tamura, R. & Saitoh, K. (2025). *Microsc. Microanal.* **31**, 1585–1586.

[bb33] Iwasaki, Y., Kimura, K. & Kitahara, K. (2023). *J. Phys. Chem. C* **127**, 20945–20950.

[bb34] Kalugin, P. & Katz, A. (2019). *Acta Cryst.* A**75**, 669–693. 10.1107/S2053273319008180 31475913

[bb35] Katz, A. & Gratias, D. (1993). *J. Non-Cryst. Solids* **153–154**, 187–195.

[bb36] Kramer, P. & Neri, R. (1984). *Acta Cryst.* A**40**, 580–587.

[bb37] Kuczera, P., Wolny, J. & Steurer, W. (2012). *Acta Cryst.* B**68**, 578–589. 10.1107/S0108768112041134 23165594

[bb38] Langsdorf, A. & Assmus, W. (1998). *J. Cryst. Growth* **192**, 152–156.

[bb39] Li, M. R., Hovmöller, S., Sun, J. L., Zou, X. D. & Kuo, K. H. (2008). *J. Alloys Compd.* **465**, 132–138.

[bb40] Lin, Q. & Corbett, J. D. (2006). *Proc. Natl Acad. Sci. USA* **103**, 13589–13594. 10.1073/pnas.0605954103 PMC1564238 16950873

[bb41] Lubensky, T. C., Socolar, E. S., Steinhardt, P. J., Bancel, P. A. & Heiney, P. A. (1986). *Phys. Rev. Lett.* **57**, 1440–1443. 10.1103/PhysRevLett.57.1440 10033450

[bb42] Lück, R. (1990). *J. Non-Cryst. Solids* **117–118**, 820–823.

[bb43] Luo, Z. P., Zhang, S. Q., Tang, Y. L. & Zhao, D. S. (1993). *Scr. Metall. Mater.* **28**, 1513–1518.

[bb44] Niikura, A., Tsai, A. P., Inoue, A. & Masumoto, T. (1994*a*). *Jpn. J. Appl. Phys.* **33**, L1538–L1541.

[bb45] Niikura, N., Tsai, A. P., Inoue, A. & Masumoto, T. (1994*b*). *Philos. Mag. Lett.* **69**, 351–355.

[bb46] Ogawa, T. (1985). *J. Phys. Soc. Jpn* **54**, 3205–3208.

[bb47] Palatinus, L. & Chapuis, G. (2007). *J. Appl. Cryst.* **40**, 786–790.

[bb48] Quiquandon, M. & Gratias, D. (2014). *C. R. Phys.* **15**, 18–29.

[bb49] Sato, T. J. (2005). *Acta Cryst.* A**61**, 39–50. 10.1107/S0108767304026625 15613752

[bb50] Sato, T. J., Abe, E. & Tsai, A. P. (1997). *Jpn. J. Appl. Phys. Lett.* **36**, L1038.

[bb51] Singh, A., Abe, E. & Tsai, A. P. (1998). *Philos. Mag. Lett.* **77**, 95–104.

[bb52] Steurer, W. (2006). *Philos. Mag.* **86**, 1105–1113.

[bb53] Strzalka, R. & Wolny, J. (2014). *Acta Phys. Pol. A* **126**, 585–587.

[bb54] Strzalka, R., Buganski, I. & Wolny, J. (2015). *Acta Cryst.* A**71**, 279–290. 10.1107/S2053273315001473 25921496

[bb55] Strzalka, R., Buganski, I. & Wolny, J. (2016). *Crystals* **6**, 104.

[bb56] Takakura, H., Gómez, C. P., Yamamoto, A., De Boissieu, M. & Tsai, A. P. (2007). *Nat. Mater.* **6**, 58–63. 10.1038/nmat1799 17160006

[bb57] Takakura, H. & Yamamoto, A. (2007). *Philos. Mag.* **87**, 2713–2720.

[bb58] Tamura, R., Abe, T., Yoshida, S., Shimozaki, Y., Suzuki, S., Ishikawa, A., Labib, F., Avdeev, M., Kinjo, K., Nawa, K. & Sato, T. J. (2025). *Nat. Phys.* **21**, 974–979.

[bb59] Tamura, R., Ishikawa, A., Suzuki, S., Kotajima, T., Tanaka, Y., Seki, T., Shibata, N., Yamada, T., Fujii, T., Wang, Ch.-W., Avdeev, M., Nawa, K., Okuyama, D. & Sato, T. J. (2021). *J. Am. Chem. Soc.* **143**, 19938–19944. 10.1021/jacs.1c09954 PMC8640986 34786934

[bb60] Tsai, A. P., Niikura, A., Inoue, A., Masumoto, T., Nishida, Y., Tsuda, K. & Tanaka, M. (1994). *Philos. Mag. Lett.* **70**, 169–175.

[bb61] Uhrig, E., Brühne, S., Assmus, W., Grüner, D. & Kreiner, G. (2005). *J. Cryst. Growth* **275**, e1987–e1991.

[bb62] Uhrig, E., Brühne, S., Sterzel, R., Schröpfer, L. & Assmus, W. (2003). *Philos. Mag. Lett.* **83**, 265–277.

[bb63] Wolny, J., Buganski, I., Kuczera, P. & Strzalka, R. (2016). *J. Appl. Cryst.* **49**, 2106–2115. 10.1107/S160057671601637X PMC5139996 27980514

[bb64] Yamada, T., Takakura, H., de Boissieu, M. & Tsai, A.-P. (2017). *Acta Cryst.* B**73**, 1125–1141.

[bb65] Yamada, T., Takakura, H., Euchner, H., Pay Gómez, C., Bosak, A., Fertey, P. & de Boissieu, M. (2016*a*). *IUCrJ* **3**, 247–258. 10.1107/S2052252516007041 PMC4937780 27437112

[bb66] Yamada, T., Takakura, H., Kong, T., Das, P., Jayasekara, W. T., Kreyssig, A., Beutier, G., Canfield, P. C., de Boissieu, M. & Goldman, A. I. (2016*b*). *Phys. Rev. B* **94**, 060103.

[bb67] Yamada, T., Takakura, H. & Yamamoto, A. (2024). *Acta Cryst.* A**80**, 422–438. 10.1107/S2053273324008568 PMC11532924 39344442

[bb68] Yamamoto, A. (1996). *Acta Cryst.* A**52**, 509–560.

