# Supplementary material for: The Ammann–Kramer–Neri tiling model of a P-ZnMgEr Bergman-type quasicrystal based on in-house X-ray diffraction
Source: Acta Crystallogr B Struct Sci Cryst Eng Mater. 2026 Feb 10;82(Pt 2):175–85. doi: 10.1107/S205252062600003X (PMC13058901; doi:10.1107/S205252062600003X)
Supplement: Supplementary file 3 [file b-82-00175-sup3.pdf]

STRUCTURAL SCIENCE
CRYSTAL ENGINEERING
MATERIALS

Volume 82 (2026)

Supporting information for article:

The Ammann-Kramer-Neri tiling model of P-ZnMgEr Bergman-type quasicrystal based on in-house X-ray diffraction

Ireneusz Buganski, Stanislav Vrtnik, Andreja Jelen, Jože Luzar, Radoslaw Strzalka, Janusz Wolny, Joachim Kusz and Nobuhisa Fujita

Table S1 The list of atoms in the asymmetric part of PR (L) and OR (S). The atomic coordinates are expressed in the reduced coordinate system, where the vector $\mathbf{r}$ of the atom is given by $\mathbf{r} = x\mathbf{d}_1 + y\mathbf{d}_2 + z\mathbf{d}_6$ for PR and $\mathbf{r} = x\mathbf{d}_1 - y\mathbf{d}_6 - z\mathbf{d}_3$ for OR, where $\mathbf{d}_1$, $\mathbf{d}_2$, $\mathbf{d}_3$ and $\mathbf{d}_6$ are vectors of the icosahedral setting. Other parameters are: $p(\mathrm{Zn})$ – probability of occupation by Zn, $p(\mathrm{Mg})$ – probability of occupation by Mg, $p(\mathrm{Er})$ – probability of occupation by Er, SOF – Site Occupancy Factor, ADP – phononic atomic displacement parameter (B factor). Fraction is the occupancy given by the special symmetry positions in rhombohedral units. Symbol L corresponds to a prolate rhombohedron, whereas S corresponds to an oblate rhombohedron.

Label	Constraining atom	x	y	z	$p(\mathrm{Zn})$	$p(\mathrm{Mg})$	$p(\mathrm{Er})$	SOF	ADP	Fraction
1L		0.000	0.000	0.000	0.000	0.000	1.000	1	1.794	0.008
2L	77L	0.232	0.000	0.000	0.000	0.000	1.000	1	1.121	0.100
3L	68L	0.383	0.000	0.000	1.000	0.000	0.000	1	1.332	0.100
4L	53L	0.487	0.000	0.000	1.000	0.000	0.000	1	7.731	0.100
5L	43L	0.606	0.000	0.000	1.000	0.000	0.000	1	8.483	0.100
6L	26L	0.747	0.000	0.000	0.000	0.000	1.000	1	3.998	0.100
7L	86L	0.854	0.000	0.000	0.000	1.000	0.000	1	4.903	0.100
8L	1L	1.000	0.000	0.000	0.000	0.000	1.000	1	1.794	0.060
9L		0.080	0.080	0.080	0.000	1.000	0.000	1	1.916	0.500
10L		0.268	0.057	0.071	1.000	0.000	0.000	1	4.910	0.500
11L		0.476	0.042	0.072	1.000	0.000	0.000	1	6.418	0.500
12L		0.389	0.000	0.127	1.000	0.000	0.000	1	5.766	0.500
13L		0.619	0.000	0.117	1.000	0.000	0.000	1	1.226	0.500
14L		0.808	0.000	0.117	0.400	0.600	0.000	1	3.128	0.500
15L		0.714	0.089	0.089	0.000	1.000	0.000	1	0.386	0.500
16L		0.153	0.009	0.158	0.000	1.000	0.000	1	0.157	0.250
17L		0.929	0.081	0.117	0.000	1.000	0.000	1	4.171	0.500
18L		0.253	0.133	0.164	1.000	0.000	0.000	1	6.351	0.500

19L		0.378	0.084	0.201	1.000	0.000	0.000	1	1.926	0.500
20L		0.522	0.144	0.144	0.000	0.000	1.000	1	0.734	0.500
21L		0.499	0.000	0.229	0.399	0.601	0.000	1	5.059	0.500
22L		0.628	0.000	0.232	1.000	0.000	0.000	1	5.661	0.500
23L		0.147	0.147	0.147	1.000	0.000	0.000	1	0.952	0.167
24L		0.758	0.145	0.119	0.000	0.000	1.000	1	8.148	0.500
25L		0.419	0.000	0.259	0.000	1.000	0.000	1	6.170	0.500
26L		1.000	0.000	0.253	0.000	0.000	1.000	1	3.998	0.150
27L		0.236	0.000	0.236	0.875	0.000	0.125	1	1.242	0.250
28L		0.744	0.000	0.250	0.000	1.000	0.000	1	1.527	0.500
29L		0.620	0.120	0.234	1.000	0.000	0.000	1	0.243	1.000
30L		0.847	0.099	0.253	1.000	0.000	0.000	1	1.245	0.500
31L		0.297	0.071	0.280	0.783	0.217	0.000	1	2.430	1.000
32L		0.381	0.156	0.265	1.000	0.000	0.000	1	0.770	1.000
33L		0.501	0.076	0.310	1.000	0.000	0.000	1	0.716	1.000
34L	22L	0.768	0.000	0.372	1.000	0.000	0.000	1	5.661	0.500
35L		0.204	0.207	0.239	0.000	0.000	1.000	1	7.203	0.167
36L		0.476	0.195	0.223	1.000	0.000	0.000	1	7.722	0.500
37L		0.607	0.247	0.237	1.000	0.000	0.000	1	1.618	0.500
38L		0.750	0.232	0.233	1.000	0.000	0.000	1	4.071	0.371
39L		0.693	0.135	0.304	1.000	0.000	0.000	1	4.232	1.000
40L		0.391	0.000	0.380	0.000	0.000	1.000	1	6.859	0.250
41L		0.622	0.000	0.380	0.000	0.000	1.000	1	3.615	0.500
42L	13L	0.883	0.000	0.381	1.000	0.000	0.000	1	1.226	0.500
43L		1.000	0.000	0.394	1.000	0.000	0.000	1	8.483	0.150
44L		0.376	0.155	0.376	1.000	0.000	0.000	1	0.754	0.500

45L		0.520	0.158	0.386	0.000	1.000	0.000	1	0.914	1.000
46L		0.785	0.156	0.387	0.000	0.000	1.000	1	8.918	1.000
47L		0.448	0.024	0.484	1.000	0.000	0.000	1	5.803	0.250
48L		0.297	0.297	0.297	0.000	1.000	0.000	1	0.422	0.167
49L		0.497	0.310	0.310	1.000	0.000	0.000	1	0.736	0.500
50L		0.649	0.074	0.451	1.000	0.000	0.000	1	1.221	1.000
51L		0.882	0.074	0.456	1.000	0.000	0.000	1	0.937	1.000
52L	21L	0.771	0.000	0.501	0.399	0.623	0.000	1	5.059	0.500
53L		1.000	0.000	0.513	1.000	0.000	0.000	1	7.731	0.150
54L		0.612	0.250	0.381	1.000	0.000	0.000	1	4.871	1.000
55L		0.382	0.287	0.368	1.000	0.000	0.000	1	3.813	0.500
56L	47L	0.516	0.024	0.552	1.000	0.000	0.000	1	5.803	0.500
57L	25L	0.741	0.000	0.581	0.000	1.000	0.000	1	6.170	0.500
58L		0.410	0.412	0.407	0.000	1.000	0.000	1	2.901	0.167
59L		0.502	0.390	0.368	1.000	0.000	0.000	1	3.598	0.500
60L		0.605	0.385	0.380	1.000	0.000	0.000	1	2.545	0.371
61L		0.452	0.270	0.452	1.000	0.000	0.000	1	0.750	0.500
62L		0.687	0.266	0.455	1.000	0.000	0.000	1	0.842	1.000
63L		0.526	0.150	0.526	1.000	0.000	0.000	1	2.174	0.500
64L		0.634	0.158	0.518	1.000	0.000	0.000	1	3.538	1.000
65L		0.768	0.150	0.526	1.000	0.000	0.000	1	1.857	1.000
66L	40L	0.620	0.000	0.609	0.000	0.000	1.000	1	6.859	0.250
67L	12L	0.873	0.000	0.611	1.000	0.000	0.000	1	5.766	0.500
68L		1.000	0.000	0.617	1.000	0.000	0.000	1	1.332	0.150
69L		0.727	0.054	0.615	0.851	0.149	0.000	1	5.054	1.000
70L		0.527	0.290	0.527	0.000	1.000	0.000	1	3.677	0.500

71L		0.768	0.141	0.641	1.000	0.000	0.000	1	1.198	1.000
72L		0.881	0.072	0.692	1.000	0.000	0.000	1	1.216	1.000
73L		0.607	0.380	0.509	1.000	0.000	0.000	1	5.089	0.500
74L		0.449	0.495	0.424	0.821	0.000	0.179	1	4.625	0.167
75L		0.625	0.233	0.618	0.000	0.000	1.000	1	0.135	0.500
76L		0.764	0.000	0.764	0.875	0.000	0.145	1	1.242	0.250
77L		1.000	0.000	0.768	0.000	0.000	1.000	1	1.121	0.150
78L		0.767	0.144	0.767	1.000	0.000	0.000	1	0.320	0.500
79L		0.842	0.009	0.847	0.000	1.000	0.000	1	0.157	0.250
80L		1.000	0.000	0.873	0.000	1.000	0.000	1	8.284	0.150
81L		0.598	0.377	0.639	1.000	0.000	0.000	1	0.190	0.129
82L		0.690	0.276	0.690	1.000	0.000	0.000	1	3.533	0.500
83L		0.927	0.072	0.880	0.000	1.000	0.000	1	0.330	0.500
84L		1.000	0.000	1.000	0.000	0.000	1.000	1	1.794	0.015
85L		0.127	0.000	0.000	0.000	1.000	0.000	1	8.284	0.100
86L		1.000	0.000	0.146	0.000	1.000	0.000	1	4.903	0.150
87L		0.883	0.000	0.192	0.400	0.593	0.000	1	3.128	0.500
1S	1L	1.000	0.000	0.000	0.000	0.000	1.000	1	1.794	0.013
2S	4S	1.000	0.000	0.232	0.000	0.000	1.000	1	1.121	0.050
3S	7S	1.000	0.000	0.383	1.000	0.000	0.000	1	1.332	0.050
4S	2L	0.768	0.000	0.000	0.000	0.000	1.000	1	1.121	0.200
5S	16L	0.847	0.009	0.158	0.000	1.000	0.000	1	0.157	0.500
6S	15S	1.000	0.000	0.487	1.000	0.000	0.000	1	7.731	0.050
7S	3L	0.617	0.000	0.000	1.000	0.000	0.000	1	1.332	0.200
8S		0.800	0.121	0.195	1.000	0.000	0.000	1	2.407	1.000
9S	12 S	0.873	0.000	0.389	1.000	0.000	0.000	1	5.766	0.500

10S	22S	1.000	0.000	0.606	1.000	0.000	0.000	1	8.483	0.050
11S	27L	0.764	0.000	0.236	0.875	0.000	0.125	1	1.242	0.500
12S	12L	0.611	0.000	0.127	1.000	0.000	0.000	1	5.766	0.500
13S	19S	0.741	0.000	0.419	0.000	1.000	0.000	1	6.170	0.500
14S	35S	1.000	0.000	0.747	0.000	0.000	1.000	1	3.998	0.050
15S	4L	0.513	0.000	0.000	1.000	0.000	0.000	1	7.731	0.200
16S		0.777	0.194	0.211	1.000	0.000	0.000	1	5.203	0.500
17S		0.806	0.125	0.389	0.469	0.531	0.000	1	4.049	1.000
18S	30S	0.883	0.000	0.619	1.000	0.000	0.000	1	1.226	0.500
19S	25L	0.581	0.000	0.259	0.000	1.000	0.000	1	6.170	0.500
20S	42S	1.000	0.000	0.854	0.000	1.000	0.000	1	4.903	0.050
21S	29S	0.771	0.000	0.499	0.399	0.601	0.000	1	5.059	0.500
22S	5L	0.394	0.000	0.000	1.000	0.000	0.000	1	8.483	0.200
23S		0.567	0.149	0.181	1.000	0.000	0.000	1	5.963	1.000
24S		0.763	0.256	0.360	0.654	0.346	0.000	1	4.805	0.500
25S	40L	0.609	0.000	0.380	0.000	0.000	1.000	1	6.859	0.500
26S	36S	0.768	0.000	0.628	1.000	0.000	0.000	1	5.661	0.500
27S	46S	0.883	0.000	0.808	0.400	0.600	0.000	1	3.128	0.500
28S	55S	1.000	0.000	1.000	0.000	0.000	1.000	1	1.794	0.012
29S	21L	0.501	0.000	0.229	0.399	0.601	0.000	1	5.059	0.500
30S	12L	0.381	0.000	0.117	1.000	0.000	0.000	1	1.226	0.500
31S		0.743	0.244	0.501	0.440	0.560	0.000	1	3.043	0.500
32S		0.749	0.114	0.598	1.000	0.000	0.000	1	5.940	1.000
33S	48S	0.750	0.000	0.744	0.000	1.000	0.000	1	1.527	0.250
34S		0.504	0.194	0.194	1.000	0.000	0.000	1	1.908	0.500
35S	6L	0.253	0.000	0.000	0.000	0.000	1.000	1	3.998	0.200

36S	22L	0.372	0.000	0.232	1.000	0.000	0.000	1	5.661	0.500
37S		0.640	0.230	0.398	1.000	0.000	0.000	1	6.017	1.000
38S		0.555	0.103	0.429	1.000	0.000	0.000	1	3.384	1.000
39S		0.709	0.204	0.596	1.000	0.000	0.000	1	3.634	0.500
40S	45S	0.620	0.000	0.622	0.000	0.000	1.000	1	3.615	0.250
41S	47L	0.552	0.024	0.484	1.000	0.000	0.000	1	5.803	0.500
42S	7L	0.146	0.049	0.048	0.000	1.000	0.000	1	4.903	0.200
43S		0.395	0.157	0.245	0.000	1.000	0.000	1	5.038	1.000
44S		0.654	0.407	0.335	1.000	0.000	0.000	1	3.832	0.276
45S	41L	0.378	0.000	0.380	0.000	0.000	1.000	1	3.615	0.250
46S	14L	0.192	0.000	0.117	0.400	0.600	0.000	1	3.128	0.500
47S		0.510	0.193	0.423	1.000	0.000	0.000	1	4.432	1.000
48S	28L	0.256	0.000	0.250	0.000	1.000	0.000	1	1.527	0.250
49S		0.578	0.379	0.469	1.000	0.000	0.000	1	3.989	0.500
50S		0.614	0.230	0.614	1.000	0.000	0.000	1	1.017	0.224
51S		0.268	0.174	0.167	1.000	0.000	0.000	1	5.770	0.500
52S		0.489	0.382	0.382	1.000	0.000	0.000	1	3.240	0.500
53S		0.428	0.316	0.474	0.219	0.781	0.000	1	3.097	0.500
54S		0.595	0.347	0.624	1.000	0.000	0.000	1	2.799	0.224
55S	8L	0.000	0.000	0.000	0.000	0.000	1.000	1	1.794	0.058
56S		0.387	0.433	0.383	1.000	0.000	0.000	1	2.747	0.167
57S	58S	1.000	0.000	0.127	0.000	1.000	0.000	1	8.284	0.250
58S	85L	0.873	0.000	0.000	0.000	1.000	0.000	1	8.284	0.200
59S		0.841	0.238	0.841	0.000	1.000	0.000	1	0.550	0.224

Table S2 The atomic composition in selected parts of the sample investigated with EDS. Points on the sample are shown in Figure S1.

Label	Mg [%]	Zn [%]	Er [%]	Total [%]
Spectrum 17	21.07	70.42	8.51	100.00
Spectrum 18	21.02	70.21	8.77	100.00
Spectrum 19	21.12	70.18	8.71	100.00
Spectrum 20	20.14	70.83	9.04	100.00
Spectrum 21	20.06	70.90	9.04	100.00
Spectrum 22	21.23	70.12	8.65	100.00
Spectrum 23	20.46	70.86	8.68	100.00
Spectrum 24	19.74	71.25	9.00	100.00
Spectrum 25	19.17	71.82	9.02	100.00
Spectrum 26	19.14	71.72	9.14	100.00

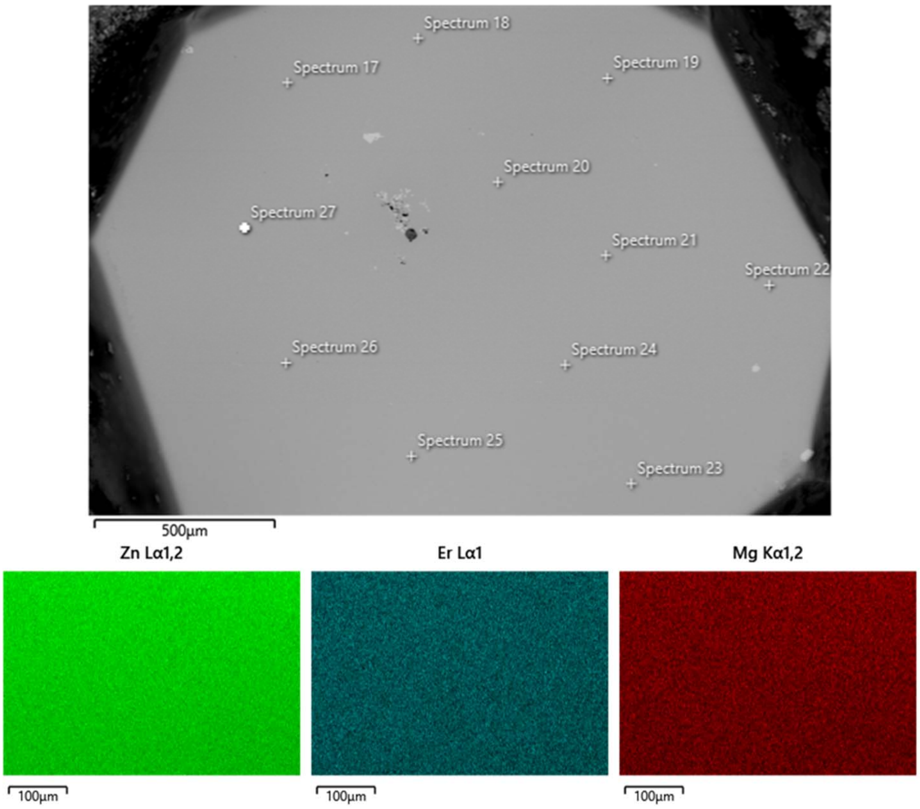

Figure S1 (Top) The points of atomic composition measurement as shown in Table S2. (Bottom) The distribution of elements in the sample.
